# Spatial ploidy inference using quantitative imaging

**DOI:** 10.1016/j.crmeth.2025.101249

**Published:** 2025-12-04

**Authors:** Nicholas J. Russell, Paulo B. Belato, Lilijana Sarabia Oliver, Archan Chakraborty, Adrienne H.K. Roeder, Donald T. Fox, Pau Formosa-Jordan

**Affiliations:** 1 Department of Plant Developmental Biology, Max Planck Institute for Plant Breeding Research, 50829 Cologne, Germany; 2 Department of Pharmacology and Cancer Biology, Duke University, Durham, NC, USA; 3 Weill Institute for Cell and Molecular Biology, Cornell University, Ithaca, NY 14853, USA; 4 School of Integrative Plant Science, Section of Plant Biology, Cornell University, Ithaca, NY 14853, USA; 5 Cluster of Excellence on Plant Sciences (CEPLAS), Max Planck Institute for Plant Breeding Research, Cologne, Germany; 6 Polyploidy Integration and Innovation Institute

**Keywords:** ploidy, endopolyploidy, endoreduplication, machine learning, image analysis, pattern formation, *Arabidopsis*, *Drosophila*, cardiomyocytes, confocal microscopy

## Abstract

Polyploidy (whole-genome duplication) is a common yet under-surveyed property of tissues across multicellular organisms. Polyploidy plays a critical role during tissue development, following acute stress, and during disease progression. Common methods to reveal polyploidy involve either destroying tissue architecture by cell isolation or tedious identification of individual nuclei in intact tissue. Therefore, there is a critical need for rapid and high-throughput ploidy quantification using images of nuclei in intact tissues. Here, we present iSPy (inferring Spatial Ploidy), an unsupervised learning pipeline that is designed to create a spatial map of nuclear ploidy across a tissue of interest. We demonstrate the use of iSPy in *Arabidopsis*, *Drosophila*, and human tissue. iSPy can be adapted for a variety of tissue preparations, including whole mount and sectioned. This high-throughput pipeline will facilitate rapid and sensitive identification of nuclear ploidy in diverse biological contexts and organisms.

## Highlights

• iSPy is a computational pipeline to generate spatial ploidy maps across a tissue
• iSPy rapidly and accurately quantifies nuclear ploidy in intact tissues
• We apply the method to *Arabidopsis*, *Drosophila*, and human nuclei
• The spatial ploidy maps reveal insights into ploidy patterning across a tissue

## Motivation

Ploidy, the number of chromosome copies in a nucleus, can vary spatially throughout a tissue, and this variation plays a critical role in eukaryotic organisms during tissue development, following acute stress, and during disease progression. However, common methods to reveal nuclear ploidy destroy the tissue and lose spatial ploidy information. As the importance of ploidy in tissue biology is increasingly recognized, powerful methods are needed to quantify nuclear ploidy throughout a tissue of interest at a given time and position without destroying tissue architecture. Therefore, we developed a high-throughput computational pipeline using quantitative image analysis from microscopy images to infer nuclear ploidy while retaining spatial information.

## Graphical abstract

**Figure:**
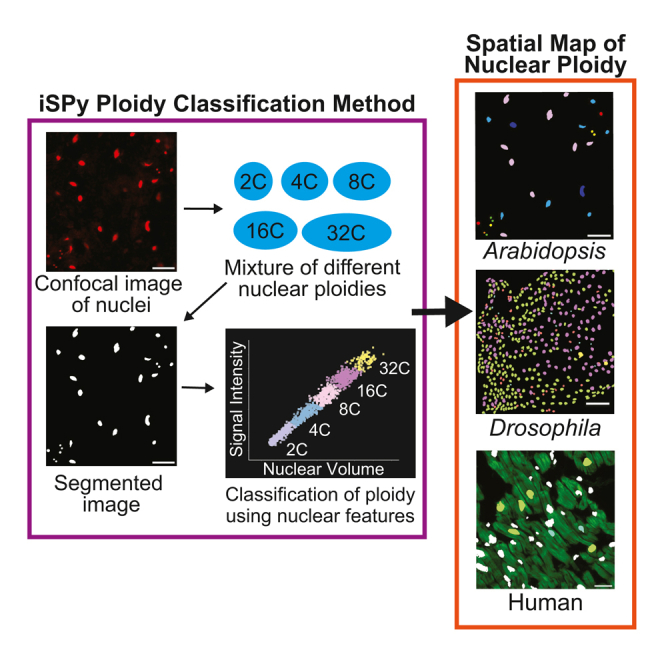

## Teaser

Russell et al. construct a high-throughput experimental and computational pipeline that calculates nuclear ploidy from microscopy images and outputs a representative ploidy map showing the spatial arrangement of the nuclear ploidies. They demonstrate and validate their pipeline using *Arabidopsis*, *Drosophila*, and human tissue.

## Introduction

Polyploidy, whereby cells have more than two homologous copies of their chromosomes, can either occur in every somatic cell of an organism (i.e., organismal polyploidy) or a subset of cells (i.e., endoploidy or endopolyploidy).^1^ Endopolyploidy commonly arises through repeated cell cycles where DNA replication occurs without completed cell division (referred to by many names, such as endoreplication) or by cell-to-cell fusion.^2^ Endopolyploid tissues are widespread among eukaryotic organisms and can be spatially patterned in both plants^3^^,^^4^^,^^5^ and animals.^6^^,^^7^^,^^8^ Endopolyploidy can occur during organ formation,^4^^,^^7^^,^^9^ or it can be promoted through external stimuli, for example, to regenerate tissue after injuries or aid in immune response.^1^^,^^10^^,^^11^ New examples of endopolyploidy are continually being identified, revealing that polyploidy is a common intrinsic property of most tissue types. However, ectopic occurrences of endopolyploidy can elevate the risks of genome instability and diseases such as cancer.^2^^,^^12^^,^^13^^,^^14^ Therefore, identifying abnormal endopolyploid cells in tissue biopsy samples is often crucial.

Given the ever-increasing appreciation of the importance of endopolyploidy in tissue biology, powerful methods should be available to accurately quantify nuclear ploidy in a tissue of interest at a given time and position. Preferably, this quantification should occur while the tissue or organism is still intact, to reveal the position of the polyploid cells. Flow cytometry is a frequently used approach to measure the ploidy distribution in tissues in both plants^15^^,^^16^^,^^17^^,^^18^ and animals.^19^^,^^20^ However, this method is invasive, and the sampled tissue is destroyed in the process, making it very difficult to recover positional information. Computational techniques have been developed to infer ploidy from sequencing data, but this also removes spatial information and is a relatively low-throughput method.^21^^,^^22^^,^^23^^,^^24^

Alternatively, high-resolution microscopy followed by single-cell analysis can give accurate measurements of ploidy while retaining spatial context.^25^^,^^26^^,^^27^ However, manual analyses of nuclear ploidy from microscopy imaging data are tedious and low-throughput, because each nucleus is measured individually. Other common methods of labeling DNA content while using fluorescence microscopy techniques include fluorescence *in situ* hybridization^28^ or DNA stains such as propidium iodide (PI), Hoechst, or 4′,6-diamidino-2-phenylindole (DAPI).^9^^,^^17^^,^^29^^,^^30^^,^^31^ Pipelines have also been created to streamline the quantification of ploidy from microscopy images, some using deep learning methods.^9^^,^^17^^,^^32^ However, these methods have not yet achieved high spatial resolution while being high-throughput, for instance, due to the large amount of background noise from PI- and DAPI-staining, necessitating laborious manual quantification of nuclear fluorescence.

In this paper, we introduce inferring spatial ploidy (iSPy), a high-throughput pipeline to quantify the ploidy of nuclei while retaining spatial information. We use confocal imaging techniques that preserve tissues and an unsupervised Gaussian mixture model to predict the ploidy of all nuclei and to produce a ploidy spatial map. We demonstrate the efficacy of this technique using three distinct model systems. First, we highlight the utility of iSPy to pinpoint developmentally programmed endopolyploidy in plant tissue: the cotyledons of *Arabidopsis thaliana* (hereafter *Arabidopsis*). We verify that iSPy-derived data from this tissue closely match data from flow cytometry. Second, we show that iSPy can track regeneration-induced endopolyploidy in the *Drosophila melanogaster* (hereafter *Drosophila*) hindgut pylorus. Third, we highlight the ability of iSPy to track ploidy differences in physically sectioned samples from human organ donor hearts. Our data reveal the broad applicability of iSPy and its ability to identify complex spatial positioning of cells with different ploidy. This allows researchers across several fields to accurately and rapidly quantify ploidy across tissues in diverse organisms and enhances the ability to identify and analyze endopolyploid cells.

## Results

### A machine-learning-based image analysis method to determine nuclear ploidy

**Figure 1:**
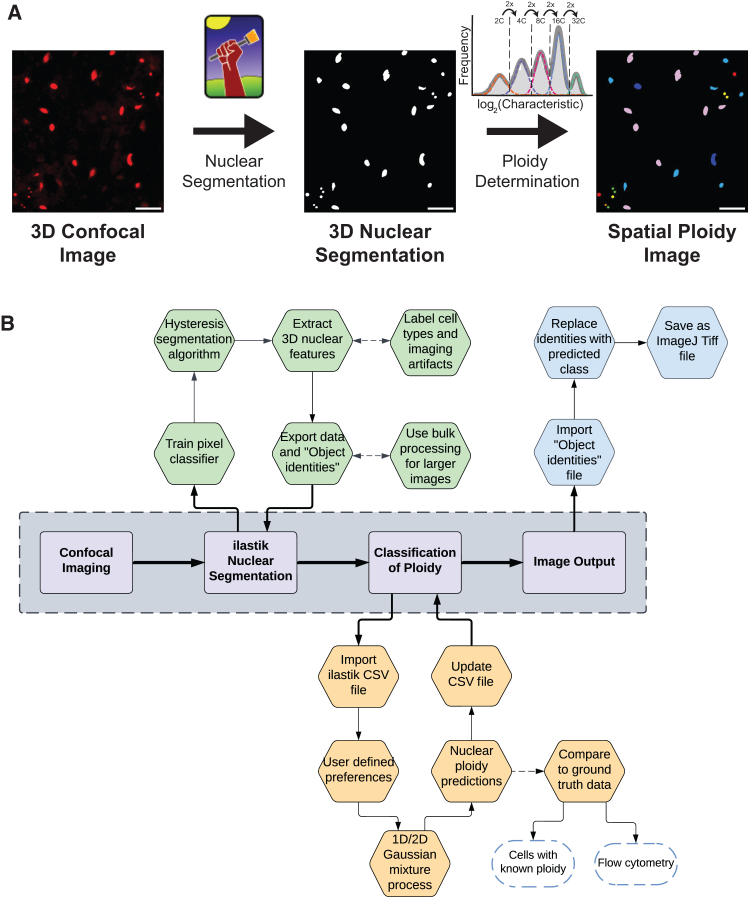
An image-based pipeline to determine spatial ploidy (A) The iSPy pipeline at a glance. Confocal imaging is performed with nuclear reporters and stains, nuclear segmentation with ilastik is conducted, and a Gaussian mixture model (Gaussian mixture) is used to classify the ploidy of each nucleus with easy-to-use software. Colors in the right panel represent different ploidy levels. Scale bars, 25 μm. (B) A more detailed overview of the iSPy procedure. See the Results and STAR Methods sections for further details. Dashed arrows indicate optional processes, which are not essential for using iSPy. See Figure S1 for a more detailed procedure for the Gaussian mixture process.

The starting point for the iSPy pipeline is a tissue preparation containing a fluorescence marker or dye that correlates with DNA content (Figure 1A). As discussed in later sections, our technique applies to a wide variety of tissue preparations from diverse organisms. From each tissue preparation, we acquired a three-dimensional confocal image with appropriate nuclear markers. We segmented the nuclei from either the raw three-dimensional confocal image or from the two-dimensional sum-projected image to obtain key nuclear features using the high-throughput image segmentation software ilastik. Ilastik lets users interactively segment nuclei using supervised classification and thresholding (see STAR Methods and subsequent subsections).^33^ Although there are many other segmentation software programs available, to illustrate our methodology, ilastik was utilized due to its low learning curve, applicability across organisms, and ease of exporting segmentations to construct a ploidy map (see subsequent subsections). For a system such as *Arabidopsis*, three-dimensional segmentation is preferred due to the presence of multiple cell types at distinct depths within the tissue. However, for *Drosophila* and human cardiomyocytes, two-dimensional segmentation of the sum-projected images is feasible due to sample preparation techniques. After nuclear segmentation was completed, we manually labeled relevant objects, such as known cell types and erroneous nuclear segmentations, using ilastik’s object classification tool. Lastly, we extracted nuclear features such as the intensity of the corresponding nuclear markers and the nuclear volume (Figures 1B and S1).

To identify clusters of nuclei with equal ploidy, we utilized an unsupervised Gaussian mixture model.^34^ This method assumes that the features we are interested in come from the combination of several normally distributed populations, and these populations correspond to the different ploidy classes (1C corresponding to the haploid genome, 2C corresponding to a diploid genome, 4C, 8C, etc.). One of the key features we were interested in was the total intensity of the nuclear markers. Because we expect the nuclear marker signal to double as genome copy-number doubles (e.g., if 2C nuclei have a mean total intensity of 2^k^, we expect 4C nuclei to have a mean of 2^k+1^, 8C with 2^k+2^, etc.), we scaled all of our data logarithmically with base 2, similar to other ploidy quantification pipelines.^22^^,^^23^^,^^30^ This scaling is performed automatically in our computational pipeline, and this pipeline outputs the best-fit model by varying the parameters of the Gaussian mixture model. To identify the Gaussian mixture model that provided the best fit for our data, we implemented well-established minimization metrics such as the Akaike information criterion (AIC) and the Bayesian information criterion (BIC) (see STAR Methods).^34^^,^^35^ We then classified each nucleus into one of the clusters by using its corresponding log likelihood probabilities (see STAR Methods).

Thus, our pipeline classifies and reports the ploidy of each nucleus within an input image and generates a two- or three-dimensional segmented image of the resulting classification, as well as data files suitable for further analyses of the spatial distribution of ploidies (Figure 1). In subsequent sections, we highlight three diverse examples of tissues where we applied our method to quantify tissue ploidy.

### iSPy facilitates ploidy determination in plant tissues expressing fluorescent reporters

In *Arabidopsis*, the ploidy level in cells of sepals, leaves, and cotyledons varies.^36^^,^^37^^,^^38^^,^^39^ Endopolyploidy is initiated at different time points throughout development, resulting in a heterogeneous ploidy distribution throughout the tissue, with cells varying from 2C to 64C.^36^^,^^40^ Previously, classification of cellular ploidy was difficult without performing flow cytometry. Recent work has demonstrated general correlations between nuclear sizes, cell sizes, and ploidy, but there are exceptions in both sepals and leaves.^36^^,^^37^ Many studies have used PI or DAPI staining to assist in calculating the nuclear ploidy of fixed tissues, although there is no standardized method to quantify ploidy from these types of images.^9^^,^^37^ Moreover, live, undamaged cells are often impermeable to PI and other dyes, meaning these stains cannot be used for single-nuclei tracking of ploidy in time-lapse microscopy of living tissues. To accurately determine the ploidy of living nuclei throughout tissue development, it is necessary to develop new techniques that do not require tissue destruction or fixation. Recently, it has been shown that the presence of histone markers in yeast correlates with genome content, but whether the same holds in plants—and in particular *Arabidopsis*—has not been explored in depth.^41^

**Figure 2:**
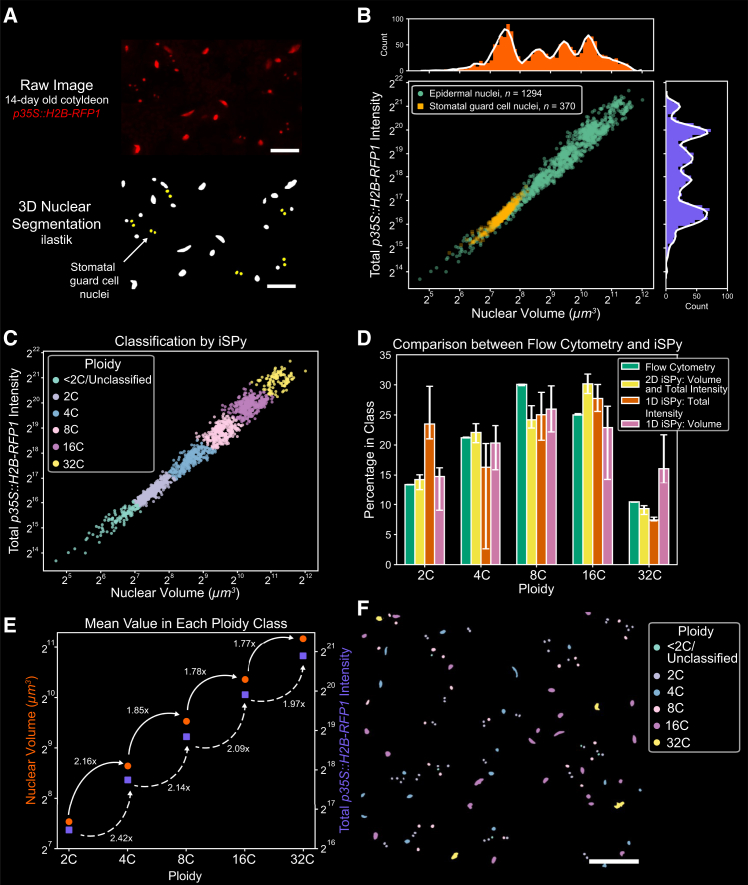
iSPy shows that the histone marker accurately quantifies ploidy in *Arabidopsis* cotyledons (A) Above: representative sum-projected confocal image of 14-day-old *Arabidopsis* cotyledons with the nuclear marker *p35S::H2B-RFP1*. Below: 3D nuclear segmentation of the above image performed in ilastik. Yellow nuclei signify hand-selected stomatal guard cell nuclei (see STAR Methods). Scale bars, 25 μm. (B) Scatterplot of the nuclear volume and total *p35S::H2B-RFP1* intensity of the ilastik-segmented epidermal nuclei (green) and stomatal guard cell nuclei (yellow), including corresponding histograms of the total *p35S::H2B-RFP1* intensity (purple) and nuclear volume (orange) with a smoothed Savitzky-Golay filter (white line, only for illustrative purposes). (C) iSPy prediction using the 2D Gaussian mixture with six components and spherical covariance matrix (see Figures S3G and S3H for additional information). (D) Comparison of the percentage of epidermal nuclei predicted in each ploidy class between flow cytometry (green, Figures S2G and S2H), the 2D Gaussian mixture with nuclear volume and total intensity (yellow, Figures 2C, S3G, and S3H), the 1D Gaussian mixture with only total intensity (red, Figures S3A–S3C), and the 1D Gaussian mixture with only nuclear volume (pink, Figures S3D–S3F). See Table S1 for exact values. Uncertainty bars represent nuclei that may be classified incorrectly (log likelihood probability less than 0.8) and nuclei that could be classified in another component (log likelihood probability greater than 0.2) (see STAR Methods). (E) The mean of the total *p35S::H2B-RFP1* nuclear intensity (purple squares, right axis) and nuclear volume (orange circles, left axis) per ploidy class with the fold increase to the next ploidy class using 2D iSPy in (C). (F) Portion of a segmented nuclear image colored with the ploidy distribution predicted from 2D iSPy in (C) across an abaxial side of a cotyledon from the same sample as in the inset shown in (A). Scale bar, 50 μm. See also Table S1.

To this end, we harvested 14-day-old cotyledons of plants expressing the fluorescently tagged nuclear histone marker (*p35S::H2B-RFP1*; Cauliflower mosaic virus promoter 35S driving expression of a histone 2B red fluorescent protein fusion) to quantify ploidy (Figure 2A; STAR Methods). We performed confocal microscopy, segmented the three-dimensional nuclei using ilastik, and hand-selected stomatal guard cell nuclei of known 2C ploidy as a standard (Figures 2A and S2A; STAR Methods).^42^ We plotted frequency histograms of the nuclear volume and the total nuclear signal intensity of each nucleus, and we observed five distinct peaks corresponding to 2C, 4C, 8C, 16C, and 32C nuclei (Figure S2B). These peaks or clusters were also apparent when considering both volume and intensity simultaneously. The stomatal guard cell nuclei, an internal standard known to be 2C, formed a cluster along with other epidermal nuclei, so we assigned this peak as 2C. However, the majority of nuclei underwent endoreduplication and were 4C and above. Note that not all fluorescent nuclear markers produce evident peaks, likely due to differences in the expression level of the promoter in different cells. Due to this variability in expression, each transgenic nuclear marker must be independently verified before extensive ploidy prediction. For example, the epidermal-specific nuclear marker *pML1::H2B-mTFP* (*Arabidopsis thaliana MERISTEM LAYER1* promoter driving expression of histone 2B fused with teal fluorescent protein) does not have evident ploidy clusters (Figures S2C and S2D). The only cluster evident contained the stomatal guard cell nuclei, which had a much lower total fluorescence intensity and volume than other epidermal nuclei, correlating with the known low level of expression of the pML1 promoter in guard cells (Figures S2C and S2D, arrow; STAR Methods).^43^ Thus, we continued to use *p35S::H2B-RFP1* in our ploidy analysis.

We compared our iSPy results with those from flow cytometry performed on 14-day-old cotyledons (Figures S2E–S2H). For flow cytometry, we utilized the epidermal-specific marker *pML1::H2B-mTFP* to distinguish epidermal from non-epidermal nuclei while simultaneously using PI staining to quantify DNA content. We observed a phenomenon similar to that in the confocal imaging data of *pML1::H2B-mTFP*, with a cluster of stomatal guard cell nuclei showing low *pML1::H2B-mTFP* fluorescence intensity (Figures S2D and S2G, arrows). Due to low *pML1::H2B-mTFP* intensity, it is possible that not all stomatal guard cell nuclei could be distinguished from non-epidermal nuclei. Therefore, to properly compare the flow cytometry data with our confocal imaging, we removed stomatal guard cell nuclei from both the confocal imaging and flow cytometry, and the sub-epidermal cells from our confocal imaging. For the flow cytometry analysis, we filtered the stomatal guard cell nuclei by setting a threshold on *pML1::H2B-mTFP* fluorescence intensity (Figure S2G, dashed line; STAR Methods). We obtained the ploidy distribution from flow cytometry by performing a Gaussian mixture using the total intensity of the PI staining in each nucleus (Figures S2G and S2H; STAR Methods). For the confocal imaging, we observed that sub-epidermal nuclei have a lower variance of intensity than epidermal nuclei, so we removed sub-epidermal nuclei by setting a threshold on the variance of intensity (Figures S2I–S2K; STAR Methods). When considering only epidermal nuclei, the peaks from the frequency histograms of nuclear volume and the total nuclear signal intensity were more evident, and the clusters when considering both nuclear features were clearer (Figure 2B).

To cluster the ploidy of epidermal nuclei from our confocal images, we compared the performance of three Gaussian mixtures: a 1D Gaussian mixture using the total *p35S::H2B-RFP1* intensity (Figures S3A–S3C), a 1D Gaussian mixture using the nuclear volume (Figures S3D–S3F), and a 2D Gaussian mixture using both of these features (Figures 2C and S3G–S3L). Six components were optimal for all three Gaussian mixtures, but they varied in their classification due to the components in which the 2C stomatal guard cell nuclei were classified. For the 1D Gaussian mixture on total intensity, the two components with the lowest mean corresponded to 2C nuclei, and the remaining four peaks corresponded to 4C, 8C, 16C, and 32C (Figures S3B and S3C). For the other two Gaussian mixtures, the component with the lowest mean corresponded to <2C/Unclassified nuclei, which were subsequently filtered out, and the other five peaks corresponded to 2C, 4C, 8C, 16C, and 32C (Figures 2C, S3E, S3F, and S3H).

The ploidy distribution of epidermal cell nuclei using the Gaussian mixture models was comparable to the ploidy distribution obtained from the flow cytometry data (Figures 2D and S3L). The 1D Gaussian mixtures, using either total nuclear signal or nuclear volume only, performed well for higher or lower ploidy levels, respectively. However, using both of these features simultaneously in the 2D Gaussian mixture allowed for an accurate classification across all ploidy levels, as shown by greater correspondence to flow cytometry results (Cramér-von Mises criterion against flow cytometry distribution: total signal, *T* = 1.642, *p* = 8.209 × 10^−5^; nuclear volume, *T* = 0.566, *p* = 0.0272; both features, *T* = 0.336, *p* = 0.10684; Figures 2D and S3L). When using the 2D Gaussian mixture model, it was calculated that the total nuclear intensity of *p35S::H2B-RFP1* increased 2.0- to 2.4-fold as ploidy doubled, whereas the nuclear volume increased by 1.8- to 2.1-fold (Figure 2E). This estimation is consistent with previous experimental and image processing work in the sepal.^37^ After classification of the ploidy of each nucleus, iSPy outputs a maximal projection image showing the spatial arrangement of nuclear ploidy in the cotyledon epidermis (Figure 2F). We observed several clusters of 4C, 8C, and 16C nuclei, while 32C nuclei were more isolated across the tissue and more likely to be farther away from stomata. Therefore, we found that the iSPy method using both total nuclear intensity and nuclear volume accurately predicts nuclear ploidy and offers a non-invasive technique to assess and visualize ploidy in leaves.

### iSPy accurately identifies injury-severity-dependent endopolyploidy in the regenerating *Drosophila* pylorus

In certain animal tissues, regeneration after injuries can occur through the induction of endopolyploid cells.^1^^,^^6^^,^^10^ Following an acute apoptotic injury in *Drosophila*, the surviving cells of the naturally diploid adult hindgut pyloric epithelium (hereafter, pylorus) endoreduplicate to restore tissue-wide DNA content to pre-injury levels.^6^^,^^44^^,^^45^ Previously, we found that the degree of ploidy is tuned to the degree of injury in the pylorus, and more specifically, the ploidy of uninjured pyloric nuclei centers around 2C, mildly injured and fully regenerated pyloric nuclei centers around 4C, and severely injured pyloric nuclei are usually 8C–16C.^44^ Regardless of the degree of injury, the final regenerated tissue-wide ploidy is similar, thus showing that endoreduplication is coordinated with the degree of cell loss.

Quantification of ploidy in these experiments has previously been performed using an established protocol, which has proven to accurately determine ploidy in various *Drosophila* tissues.^7^^,^^26^^,^^44^^,^^46^^,^^47^ Briefly, this protocol involves labeling nuclei with Hoechst to analyze DNA content and physically compressing (squashing) the pylorus, reducing the thickness and the variation in distance from the imaging objective to individual nuclei, and providing a more accurate ploidy quantification. Using this validated method, we previously detailed an accurate protocol whereby the ploidy of individual nuclei is defined and measured in Fiji.^26^ This protocol provided spatial ploidy information; however, the manual segmentation of nuclei was labor-intensive, thus prohibiting high-throughput analysis.

**Figure 3:**
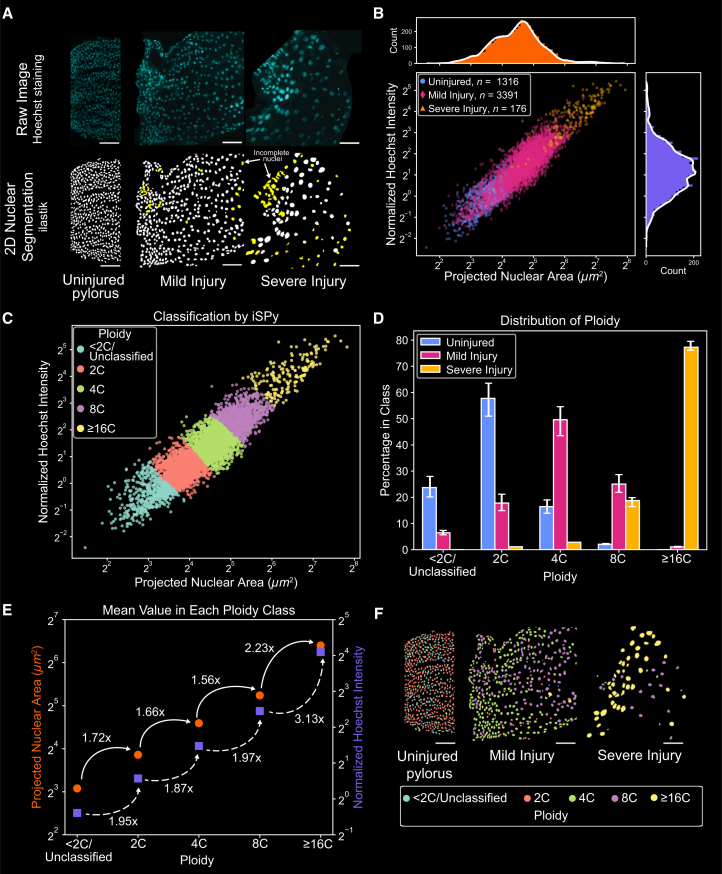
iSPy correctly predicts that *Drosophila* pyloric cells endoreduplicate in a severity-dependent manner after injury (A) Above: representative sum-projections of confocal images of pyloric nuclei stained with Hoechst (blue, see STAR Methods) from an uninjured, mildly injured, and severely injured pylorus (left to right). Below: segmented nuclei using ilastik. Yellow segmented objects denote incomplete nuclei; there were no incomplete nuclei in the uninjured pylorus. Note that this segmentation was performed with the sum projection in two dimensions. The anterior and posterior sides of the tissue axis are on the left and right sides in each image, respectively. Scale bars, 50 μm. (B) Scatterplot of the projected nuclear area and normalized Hoechst intensity of the segmented nuclei (uninjured, blue circles; mild injury, red diamonds; severe injury, yellow triangles), including corresponding histograms of the normalized Hoechst intensity (purple) and projected nuclear area (orange) with a smoothed Savitzky-Golay filter (white line, only for illustrative purposes). (C) iSPy ploidy prediction using the 2D Gaussian mixture with five components and diagonal covariance matrix (see Figure S5K for additional information). (D) The proportion of pyloric cells in each ploidy class predicted from the 2D Gaussian mixture in (C) by severity of injury (uninjured, blue; mild injury, red; severe injury, yellow). Uncertainty bars represent nuclei that may be classified incorrectly (log likelihood probability less than 0.8) and nuclei that could be classified in another component (log likelihood probability greater than 0.2) (see STAR Methods). See Table S1 for exact values. (E) The mean of the normalized Hoechst intensity (purple squares, right axis) and projected nuclear area (orange circles, left axis) with the fold increase to the next ploidy class using the 2D Gaussian mixture as shown in (C). (F) Segmented nuclear image colored with the ploidy distribution predicted from iSPy shown in (C) using the images from (A). Scale bars, 50 μm. See also Table S1.

To determine whether iSPy could accurately produce spatial ploidy maps of regenerating *Drosophila* pylori, we used an established genetic cell ablation protocol to injure the pylorus and analyze tissue ploidy.^26^^,^^44^^,^^45^^,^^47^^,^^48^^,^^49^ We examined three different conditions: uninjured pylori, mildly injured pylori (24 h at 29°C), and severely injured pylori (48 h at 29°C) (see STAR Methods). As performed previously, we used an internal control; haploid (1C) spermatids were placed on the same slide (Figure S4).^50^ We performed a sum projection of all images, segmented the projected two-dimensional nuclei in ilastik, and selected the pyloric (Figure 3A) and spermatid (Figure S4A) nuclei that were fully complete (see STAR Methods). After extracting relevant nuclear information such as total Hoechst intensity and its projected area, we normalized the total Hoechst intensity of the pyloric nuclei to the median intensity of the haploid spermatids from the same experiment to account for variability between experiments as we have done previously (Figure S4B; STAR Methods).

We observed a wide range of values for the normalized Hoechst intensity and projected nuclear area, regardless of the severity of injury (Figure 3B). Overall, the distribution of nuclei from the uninjured pylorus had a lower Hoechst intensity and projected area than those from either the mild or severe injuries. We also observed a clear distinction between the distribution of nuclei from the mildly and severely injured pylori. From previous work, we expected to identify nuclei with a ploidy between 2C and 16C.^47^

Similar to the analysis of the *Arabidopsis* data, we compared the performance of three different Gaussian mixtures: a 1D Gaussian mixture using the normalized Hoechst intensity (Figures S5A–S5E), a 1D Gaussian mixture using the projected nuclear area (Figures S5F–S5J), and a 2D Gaussian mixture using both of these features (Figures 3C and S5K–S5O). For the 1D Gaussian mixtures, we obtain different results depending on the characteristic we choose. Using only the normalized Hoechst intensity, six components were optimal when using AIC, and two components were optimal when using BIC (Figure S5A). However, using just the projected nuclear area, five components were optimal when using AIC, and four components were optimal when using BIC (Figure S5F). The projected number of components when using nuclear area is consistent with the expected number of ploidy levels in *Drosophila* pylori (2C–16C). When assessing these curves by eye, five components better fit the data than four components, which led to one very large Gaussian curve that encompassed two ploidy classes (Figures S5G–S5J, arrows). Therefore, we focused our analysis on the 1D Gaussian mixtures with five components, corresponding to the following ploidy classes: <2C/Unclassified, 2C, 4C, 8C, and ≥16C. The ≥16C class was defined as such because some nuclei had such a high concentration of normalized Hoechst intensity that they may be higher than 16C. Given that the 1D Gaussian mixture identified five components as optimal, we searched for the best fit for the 2D Gaussian mixture using one to five components. Both AIC and BIC predicted that five components provided the best fit (Figures 3C and S5K–S5M; STAR Methods). After this classification, iSPy output maximal projections showed the spatial arrangement of nuclear ploidy in the uninjured, mildly injured, and severely injured pylori (Figure 3F).

As a result of the classifications by iSPy, we found a marked difference among the ploidy distributions of the three different pylori (Figures 3D, S5C, S5H, and S5N). Our analysis of pyloric ploidy after mild and severe injury recapitulated previous results, with the majority of uninjured pyloric nuclei being 2C, the majority of mildly injured pyloric nuclei being 4C, and the majority of severely injured pyloric nuclei being ≥16C.^44^ However, iSPy showed there was a mixture of ploidy in each tissue because uninjured tissues had nuclei between 2C and 8C, but both mild and severely injured tissues had nuclei between 2C and ≥16C. We also found that the mean normalized Hoechst intensity increased 1.87- to 1.97-fold as ploidy doubled, except for the ≥16C group, which increased 3.13-fold from the 8C group, providing more evidence that there may be nuclei that are ≥16C in this group (Figures 3E and S5O). After this classification, iSPy output maximal projections showing the spatial arrangement of nuclear ploidy in the uninjured, mildly injured, and severely injured pylori (Figure 3F). These color-coded maps reveal that pyloric nuclei with increased ploidy exist throughout the anterior–posterior axis of the tissue for both mildly and severely injured cases. Therefore, we found that the iSPy method recapitulates the injury-severity-dependent endopolyploidy in the *Drosophila* pylorus while providing insights into the distributions of ploidy throughout the regenerated tissue.

### iSPy verifies tissue-specific polyploidy in human cardiomyocytes

Lastly, we assessed the ability of iSPy to analyze ploidy in tissue sections from larger organs, using the human heart as an example. The muscle cells of the heart, known as cardiomyocytes, become endopolyploid throughout the development of many organisms, making it an appropriate polyploidy system to study.^7^^,^^51^^,^^52^^,^^53^^,^^54^ Following developmental polyploidization, ploidy in the adult human myocardium remains stable.^55^ Recently, we showed that in adult human cardiomyocytes, the degree to which cardiomyocytes become polyploid during development is chamber-specific and correlates with higher levels of insulin signaling.^7^ This chamber specificity leads to higher cardiomyocyte ploidy in the adult left ventricle (LV) relative to the left atrium (LA).

**Figure 4:**
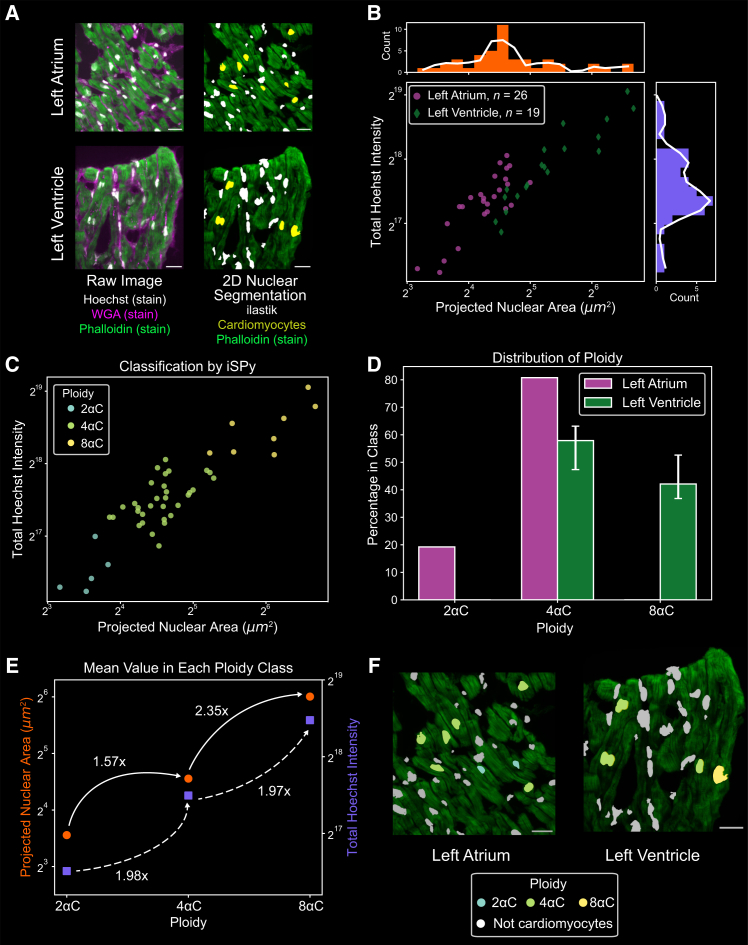
iSPy predicts three distinct ploidy classes for human cardiomyocytes (A) Left: representative sum-projections of confocal images of human heart tissue stained with Hoechst (white), wheat germ agglutinin (WGA, magenta), and phalloidin (green) from the left atrium (top) and left ventricle (bottom). Right: segmented nuclei using ilastik. Yellow nuclei signify hand-selected cardiomyocytes using phalloidin as a guide (see STAR Methods). Note that this segmentation was performed with the sum projection in two dimensions. Scale bars, 25 μm. (B) Scatterplot of the projected nuclear area and total Hoechst intensity of the segmented cardiomyocytes (left atrium, purple circles; left ventricle, green diamonds), including corresponding histograms of the normalized Hoechst intensity (purple) and projected nuclear area (orange) with a smoothed Savitzky-Golay filter (white line, only for illustrative purposes). (C) iSPy ploidy prediction using the 2D Gaussian mixture with three components and spherical covariance matrix, where 2αC, 4αC, and 8αC are relative ploidy levels (see Figure S6A for additional information). (D) The proportion of cardiomyocytes in each ploidy class predicted from the 2D Gaussian mixture in (C) by heart chamber (left atrium, purple; left ventricle, green). Uncertainty bars represent nuclei that may be classified incorrectly (log likelihood probability less than 0.8) and nuclei that could be classified in another component (log likelihood probability greater than 0.2) (see STAR Methods). See Table S1 for exact values. (E) The mean of the Hoechst intensity (purple squares, right axis) and projected nuclear area (orange circles, left axis) with the fold increase to the next ploidy class using the 2D Gaussian mixture from (C) with three components and spherical covariance matrix. (F) Segmented nuclear image colored with the ploidy distribution predicted from the 2D Gaussian mixture (C) using the images from (A). Scale bars, 25 μm. See also Table S1.

In this study, we re-analyzed confocal microscopy images of frozen heart tissue sections taken from anonymized donors (men aged between 41 and 44 years from the Duke Human Heart Repository [DHHR]; see STAR Methods). The tissues were labeled with Hoechst to analyze DNA content, wheat germ agglutinin (WGA) to mark cell membranes, and phalloidin to mark actin and imaged via fluorescence confocal microscopy (Figure 4A; STAR Methods). We performed a sum projection and segmented the resulting two-dimensional image in ilastik. Using the phalloidin labeling of cardiomyocyte muscle striations as a guide, we manually selected the cardiomyocytes from each image and extracted the total intensity of the Hoechst staining and the projected nuclear area (Figure 4A, yellow nuclei; STAR Methods).

As we found previously using individual hand-drawn nuclear segmentation, we found that LV cardiomyocytes had a larger projected nuclear area and a higher total Hoechst intensity than the LA cardiomyocytes (Figure 4B).^7^ We observed clusters with three successive genome doubling levels of relative Hoechst intensity and nuclear area, and we expected to find three classes of ploidy: 2αC, 4αC, and 8αC, where α is a positive integer. Past observations have shown that cardiomyocytes are between 2C and 8C, implying that α = 1.^51^ However, since we cannot verify the exact ploidy levels in our samples, we report the results in the heart in terms of relative ploidies, being 2αC, 4αC, and 8αC.

Due to the relatively small sample size (*n* = 26 for LA, *n* = 19 for LV), we only performed a 2D Gaussian mixture with both total Hoechst intensity and the projected nuclear area. We searched for the best fit by varying the number of components between one and three and found that three components provided the best fit for both information criteria (Figures 4C and S6A–S6C). As expected from our previous work, the ploidy proportion of LV and LA cardiomyocytes differed greatly, with LA cardiomyocytes having a ploidy of either 2αC or 4αC and LV cardiomyocytes having a ploidy of either 4αC or 8αC (Figures 4D and S6D). Moreover, our procedure accurately captured the 2-fold increase in total Hoechst intensity as ploidy increased, whereas we observed a 1.6- to 2.4-fold or 2.0- to 2.8-fold increase in the projected nuclear area as ploidy increased, depending on the covariance matrices used (Figures 4E and S6E). Following this classification, iSPy generated a maximal projection image showing the spatial arrangement of nuclear ploidy of the classified cardiomyocyte ploidy from our predictions (Figure 4F). Thus, iSPy verified a chamber-specific ploidy dependence in human cardiomyocytes using the Hoechst intensity and projected nuclear area.

## Discussion

Inferring the ploidy of nuclei within a tissue without destroying the integrity of the sample is critically important for the study of the development and growth of an organism and also for the analysis of emerging spatial patterns related to ploidy. In this paper, we introduce iSPy, a high-throughput method of measuring the ploidy of nuclei while simultaneously maintaining the spatial integrity of the tissue samples. This method uses simple experimental and segmentation tools along with an unsupervised Gaussian mixture model that uses only the size of the nuclei and the intensity of the fluorescence/staining signals to accurately identify nuclear ploidy and output a maximal projection image of the segmentation with its ploidy prediction. Table 1 summarizes the techniques used with iSPy experimentally and computationally for each model organism discussed in this work. Based on our analysis presented here, our pipeline could be applied to all tissue types in any organism, regardless of tissue properties or the need to perform tissue sectioning. The inclusion of other nuclear or cell membrane markers would allow detailed analyses regarding the effects of gene expression and cell size on ploidy. iSPy can also be effective in live-imaging and time-lapse experiments to analyze ploidy development both in time and space across a tissue.

**Table 1:** Methodology and results summary for iSPy on *Arabidopsis* cotyledons, *Drosophila* pylorus, and human heart tissue

Organism	Experiments	ilastik segmentation	iSPy analysis
*Arabidopsis thaliana*	• nuclear marker: *p35S::H2B-RFP1* • live or fixed tissue	• three-dimensional volume segmentation • hand-select stomatal guard cells using the membrane marker *pUBQ10::MYR-CFP* and remove incomplete nuclei	• remove sub-epidermal cells using a threshold for “Variance of Intensity” • 2D iSPy using nuclear volume and total *H2B* intensity: 6 components, spherical or diagonal covariance • remove nuclei in the component with the lowest mean • 2.0- to 2.4-fold increase in total intensity • 1.8-fold increase in volume
*Drosophila melanogaster*	• protocol from Clay et al.^26^ • nuclear marker: Hoechst staining	• two-dimensional segmentation using sum projection • hand-select and remove incomplete nuclei	• normalize pyloric nuclei by the median of 1C sperm nuclei • 2D iSPy using normalized Hoechst intensity and projected area: 5 components, spherical or diagonal covariance • 1.8- to 2.0-fold increase in normalized intensity • 1.6- to 1.7-fold increase in projected nuclear area
Human cardiomyocytes	• protocol from Chakraborty et al.^7^ • nuclear marker: Hoechst staining • optional staining: WGA and phalloidin to help identify cardiomyocytes	• two-dimensional segmentation using sum projection • hand-select cardiomyocytes (using phalloidin to help identify them)	• 2D iSPy using total Hoechst intensity and projected nuclear area: 3 components, spherical or diagonal covariance • 2.0-fold increase in total Hoechst intensity • 1.5- to 2.5-fold increase in projected nuclear area

See STAR Methods and Results sections for more specific details.

Some improvements can be made to this technique in the future. Other histone markers and promoters could be examined to see whether better candidates exist for ploidy quantification. However, variability in expression will necessitate that each promoter and histone marker be independently verified to ensure an accurate reflection of ploidy. Although unsupervised Gaussian mixture models can easily identify well-separated clusters, other supervised and unsupervised clustering algorithms can be tested, especially if there are systems where assuming Gaussian distributions is not possible (e.g., *k*-means and hierarchical clustering algorithms).^56^ Furthermore, these clusters might be easier to identify when more nuclear geometrical features are taken into account, and the implementation of spatial statistics such as radial distribution functions or tools from topological data analysis might allow patterns and underlying ploidy architecture to be identified.^57^

In this study, we employed ilastik as a segmentation software, but other segmentation tools are available and can be used with our software, provided that it outputs a segmentation image where all pixels belonging to an object have the value of the object’s label in a CSV file. Examples of other segmentation tools providing this kind of output include Fiji, CellPose, PlantSeg, CelloType, and Segment Anything for Microscopy, among others.^58^^,^^59^^,^^60^^,^^61^ Some of these tools can be high-throughput, but they may have a steeper learning curve than ilastik, particularly for those users who are not familiar with segmentation software. However, precision and speed should be balanced with user-friendliness when conducting segmentations, particularly for large datasets. Therefore, when considering whether to use ilastik, one should consider the number of tissue samples involved. For example, in this study in which we are limited by the number of tissue sections from human heart samples, the amount of effort did not save more time than our previous approach, where we individually identified each nucleus by eye.^7^ However, as one scales up to larger numbers of samples and images, the high-throughput capabilities of ilastik become superior to those of the individual nucleus identification approaches.

In conclusion, this *in silico* methodology opens a new avenue to assess—in a high-throughput manner—how ploidy affects nuclear size, cells, and tissues and how endopolyploidy is spatially patterned across organisms.

### Limitations of the study

When finding the optimal number of ploidy classes, AIC and BIC may have limitations to assess the viability of the number of classes. For instance, in some cases, these metrics show a monotonic decreasing behavior, and therefore, there is not a local minimum, and a maximum ploidy needs to be assumed to determine the number of classes. Therefore, whenever possible, it is advisable to make use of the prior knowledge about the studied system and have an idea of the types of ploidies one might expect in a certain tissue. Nevertheless, for organisms that do not have a “ground truth” or an *a priori* understanding of the ploidy distribution one should expect in their tissue, using AIC and BIC to find the optimal number of ploidy classes is an easy way to initially gauge their system. There are other types of information criteria/energy minimization techniques such as ABC^62^ or extended BIC^63^ that may be useful to find the optimal number of clusters, particularly with higher dimensional data.^64^ Additionally, Gaussian mixture models may not find ploidy classes with a very low representation of ploidy. In this case, other clustering algorithms such as *k*-means may be more useful.

## Resource availability

### Lead contact

Requests for further information and resources should be directed to the lead contact, Pau Formosa-Jordan (pformosa@mpipz.mpg.de).

### Materials availability

This study did not generate new unique reagents. Requests for all materials used in this study should be directed to the lead contact, Pau Formosa-Jordan (pformosa@mpipz.mpg.de).

### Data and code availability

• All data presented in the study are publicly available in the OSF data repository (https://osf.io/um7r3/; https://doi.org/10.17605/osf.io/um7r3).
• The code for iSPy can also be found in the OSF data repository (https://osf.io/um7r3/; https://doi.org/10.17605/osf.io/um7r3), as well as in a GitLab repository, https://gitlab.gwdg.de/devplantpatterning/Publications/ispy-inferring-spatial-ploidy.
• Any additional information required to reanalyze the data reported in this work is available from the lead contact upon request.

## Acknowledgments

We thank Weibing Yang for generating the line carrying the reporters *pUBQ10::MYR-CFP* ✕ *p35S::H2B-mRFP1* and kindly sharing it with us. We thank John Chandler, Franziska Turck, Jake Klemm, Chun-Biu Li, and André Marques for providing critical feedback on the manuscript. Funding for this work is acknowledged from the 10.13039/501100004189Max Planck Society for N.J.R. and P.F.-J.; the 10.13039/501100001659Deutsche Forschungsgemeinschaft (DFG, German Research Foundation) under Germany’s Excellence Strategy – EXC-2048/1 – project ID 390686111 for P.F.-J.; the Polyploidy Integration and Innovation Institute (PI3) US NSF DBI-2320251 for D.T.F., P.F.-J., and A.H.K.R.; and 10.13039/100000002NIH grant R01GM118447 for D.T.F.

## Author contributions

Conceptualization, N.J.R., P.B.B., L.S.O., P.F.-J., and D.T.F.; data curation, N.J.R., P.B.B., L.S.O., and A.C.; formal analysis, N.J.R., P.B.B., L.S.O., and A.C.; funding acquisition, P.F.-J., D.T.F., A.C., and A.H.K.R.; investigation, N.J.R., P.B.B., L.S.O., and A.C.; methodology, N.J.R., P.B.B., L.S.O., and A.C.; project administration, N.J.R., P.F.-J., and D.T.F.; resources, P.F.-J.; software, N.J.R.; supervision, P.F.-J., D.T.F., and A.H.K.R.; validation, N.J.R., P.B.B., L.S.O., and A.C.; visualization, N.J.R.; writing—original draft, N.J.R., D.T.F., P.B.B., A.C., and L.S.O.; writing—review & editing, N.J.R., P.F.-J., D.T.F., P.B.B., A.H.K.R., and L.S.O.

## Declaration of interests

The authors declare no competing interests.

## STAR★Methods

### Key resources table

**Table:** 

REAGENT or RESOURCE	SOURCE	IDENTIFIER
**Biological samples**		

Human cardiomyocytes	Chakraborty et al.^7^	IRB Approval Pro00005621

**Chemicals, peptides, and recombinant proteins**		

MgSO4 hydrate	Mallinckrodt Chemicals	Cat # 10034-99-8
KCl	Fisher Scientific	Cat # 7447-40-7
HEPES	Sigma Aldrich	Cat # 7365-45-9
Dithiothreitol (DTT)	Research Products International	Cat # 3483-12-3
Triton X-100	Amresco	Cat # 9002-93-1
Propidium iodide	Sigma Aldrich	Cat # 25535-16-4
Hoechst	Thermo Scientific	Cat # 62249
Wheat germ agglutinin	Invitrogen	Cat # W21404
Alexa Fluor 488 Phalloidin	Cell Signaling	Cat # 8878

**Deposited data**		

*A. thaliana* confocal images	This paper	Open Science Framework Data: https://doi.org/10.17605/osf.io/um7r3
*D. melanogaster* confocal images	This paper	Open Science Framework Data: https://doi.org/10.17605/osf.io/um7r3
Human cardiomyocytes confocal images	This paper	Open Science Framework Data: https://doi.org/10.17605/osf.io/um7r3
ilastik segmentation files for *A. thaliana*, *D. melanogaster,* and cardiomyocytes	This paper	Open Science Framework Data: https://doi.org/10.17605/osf.io/um7r3
*A. thaliana* flow cytometry of cotyledons	This paper	Open Science Framework Data: https://doi.org/10.17605/osf.io/um7r3

**Experimental models: organisms/strains**		

*D. melanogaster: byn >Gal4*	Singer et al.^65^	FBal0137290
*D. melanogaster: tub-Gal80*^*ts*^	McGuire et al.^66^	FBto0000151
*D. melanogaster: UAS-hid*/*TM3 Sb*	Grether et al.^67^	FBst0086295
*A. thaliana:* Col-0, *p35S::H2B-RFP1* ✕ *pUBQ10::MYR-CFP*	This paper	N/A
*A. thaliana:* Col-0, *p35S::H2B-RFP1*	Federici et al.^68^	N/A
*A. thaliana:* Col-0, *pUBQ10::MYR-CFP*	Yang et al.^69^	N/A
*A. thaliana:* Col-0*, pML1::mCitrine-RCI2A* ✕ *pML1::H2B-mTFP*	Robinson et al.^37^	ABRC stock CS73343

**Software and algorithms**		

ilastik	Berg et al.^33^	Version 1.3.3
iSPy	This paper	Deposited at https://doi.org/10.17605/osf.io/um7r3 and at https://gitlab.gwdg.de/devplantpatterning/Publications/ispy-inferring-spatial-ploidy

**Other**		

Leica Stellaris 8	Leica Microsystems	N/A
Nikon Ti2 Eclipse with a Nikon A1 camera	Nikon Instruments Inc.	N/A
Andor Dragonfly 505 system with Borealis illumination	Oxford Instruments	N/A
BD Accuri C6 flow cytometer	BD Biosciences	N/A

### Experimental model and study participant details

#### *Arabidopsis thaliana* growth conditions

For flow cytometry, seeds were sown directly onto Lambert LM-111 All Purpose Mix soil and stratified at 4°C for 2 days in darkness. Plants were grown under continuous fluorescent light (∼80 μmol m^−2^ s^−1^) at 22°C and 60–75% relative humidity. The Columbia-0 (Col-0) accession line carrying *pML1::mCitrine-RCI2A* and *pML1::H2B-mTFP* was used.^37^^,^^40^ Cotyledons were harvested 14 days after stratification.

For confocal imaging, seeds were stratified in water for 3–4 days at 4°C before being sown onto soil. Plants were grown in a Percival AR-95L3 chamber at 22°C and 60% relative humidity at 15% light (4592 Lux) in continuous light. The Col-0 accession line carrying *p35S::H2B-RFP1*^68^ and *pUBQ10::MYR-CFP*^69^ was used. This line was generated by Weibing Yang by transforming the membrane marker *pUBQ10::MYR-CFP* into Col-0 wild-type plants and afterward crossing them with Col-0 plants expressing *p35S::H2B-RFP*1.^68^^,^^69^ *pUBQ10::MYR-CFP* was generated using a similar strategy as for *pUBQ10::acyl-YFP* in Willis et al.^70^ Cotyledons were harvested 14 days after stratification.

#### *Drosophila melanogaster* experimental conditions

All female adult flies were aged for 4–7 days after eclosion before injury experiments. We only analyzed female fly pylorus material for this study to remove any confounding impacts of metabolism on injury-induced ploidy, as female flies have a higher metabolic demand due to egg production. Flies were kept at 18°C except during injury. Injury through a tissue-specific, genetic ablation system was induced in a temperature-dependent manner; flies were transferred from an 18°C to a 29°C incubator to induce injury. The class of severity of injury was determined by the duration that the flies were at 29°C; mildly injured flies were kept at 29°C for 24 h, while severely injured flies were kept at 29°C for 48 h. Flies were allowed to recover for at least 3–4 days before dissection. As described previously, *byn-Gal4*, *tub-Gal80*^*ts*^, and *UAS-hid/TM3 Sb* were used for injured and uninjured flies.^44^

#### Human cardiomyocyte experimental conditions

Human heart tissue samples from explanted hearts were obtained from the Duke Human Heart Repository (DHHR) with approval from the Duke University Health System (DUHS) Institutional Review Board (IRB) (Pro00005621).^7^ We only analyzed male heart material for this study due to limited sample availability. In our previous work, we did not notice a difference between heart chamber ploidy in male versus female samples. We analyzed five left ventricle and five left atrium samples from men between the ages of 41 and 44.

### Method details

#### Data acquisition for *Arabidopsis thaliana*

For confocal imaging, a Leica Stellaris 8 confocal microscope was used, with a NA 0.95 and 25× water dipping objective and a z-step of 0.4 μm. Cotyledons were placed onto microscopy slides with a coverslip. To detect *p35S::H2B-RFP1*, a laser with 590 nm emission wavelength and 2.0 intensity was used, and fluorescence was captured at 605–644 nm with a 45.5% gain. For *pML1::H2B-mTFP*, a laser with 462 nm emission wavelength and 2.0 intensity was used, and fluorescence was captured at 467–520 nm with a 20% gain. For *pML1::mCitrine-RCI2A*, the laser emission wavelength was 515 nm, laser intensity was set to 2.0, and fluorescence was captured at 523–556 nm with a 47.8% gain. Following acquisition, image files were stitched together inside LAS X software and then exported. The adaxial and abaxial sides of two cotyledons were imaged.

#### Flow cytometry for *Arabidopsis thaliana*

Tissue from seedlings expressing *pML1::H2B-mTFP* was harvested from 6 to 8 cotyledons per sample at 14 days post-stratification. Flow cytometry was performed as described previously.^37^^,^^68^ For each sample, harvested tissue was thoroughly chopped with a sterile razor blade in a Petri dish on ice containing 470 μL Aru buffer (10 mM MgSO4 hydrate, 50 mM KCl, 5 mM HEPES, 10 mM DTT (dithiothreitol), 2.5% v/v Triton X-100). The solution was filtered through a 40-μm Fisherbrand cell strainer and 350 μL were transferred to a 5 mL round bottom tube. Suspended nuclei were then treated with RNAse (0.1 mg/100 μL sample) and stained with PI (0.001 mg/100 μL sample). Samples were run on a BD Accuri C6 flow cytometer. Events were gated to separate epidermal (TFP-positive) nuclei from non-epidermal (TFP-negative) nuclei using the FL1 (533/30) channel. Relative nuclear DNA content was determined by PI fluorescence of epidermal and non-epidermal cells using the FL2 (585/40) channel.

#### Data acquisition for *Drosophila melanogaster*

For each injury condition, 3–4 pylori and 2 *Drosophila* testes (the sperm of which are a 1C haploid control) were placed on the same slide. We conducted 3 experiments for each injury condition, and, by using our criteria for choosing quality experiments (see Nuclear segmentation and data processing for Drosophila melanogaster), we obtained 3 uninjured, 9 mildly injured, and 5 severely injured pylori. Details of the tissue sample preparation and Hoechst staining have been previously described.^26^ Samples were imaged using a Nikon Ti2 Eclipse with a Nikon A1 camera and a NA 1.42 and 60× oil dipping objective with a z-step size of 0.4 μm. To detect Hoechst, the laser emission wavelength was 405 nm with 1.78 intensity, and fluorescence was captured at 420–480 nm with 42% gain.

#### Data acquisition for human cardiomyocytes

Details of the tissue sample preparation, immunostaining, and imaging have been previously described.^7^ Briefly, flash-frozen human left ventricular (LV) and left atrial (LA) tissue samples were sectioned at a thickness of 10 μm and immunostained with wheat germ agglutinin (WGA) (1:250, W21404; Invitrogen), Alexa Fluor 488 Phalloidin (1:250, 8878; Cell Signaling), and Hoechst. Samples were imaged using an Andor Dragonfly 505 system with Borealis illumination on a spinning-disk confocal microscope and an Andor Zyla PLUS 4.2 Megapixel sCMOS camera, using a z-step size of 0.5 μm, coupled with a 63×/1.47 TIRF HC PL APO CORR oil objective (Leica 11506319; working distance: 0.10 mm).

#### Nuclear segmentation and data processing for *Arabidopsis thaliana*

After image acquisition, images (as .lif files) were imported into Fiji (ImageJ) and exported as HDF5 files (.h5) using the ilastik plugin. Additionally, three crops of each confocal image were exported as an HDF5 to train ilastik with. In ilastik, the “Pixel + Object Classification Workflow” was used to import the three cropped images. After conducting the pixel training between nuclei and background on all three crops, hysteresis thresholding was performed for the nuclear segmentation with smoothing parameters (1.5, 1.5, 1.5), a core threshold of 0.75, and a final threshold of 0.45, while marking the “Don’t merge objects” box. All available nuclear features were calculated, and no nuclei were classified during the “Object Classification” step. Object Predictions were exported using a 16-bit data type as an HDF5 file, as well as the CSV file for the Feature Table. Batch Processing was performed on the large file corresponding to the three individual crops, which automatically output the Object Predictions HDF5 file and the CSV file with nuclear features. Then, the “Object Classification [Inputs: Raw Data, Segmentation]” Workflow (creating a whole new ilastik file) was used, and both the raw data file and the segmentation file of the large image were imported. Stomatal guard cell nuclei were selected with the aid of the membrane marker *pUBQ10::MYR-CFP*,^70^ which identifies the stomatal cells clearly (Figure S2A). Nuclei that were incorrectly segmented, merged with another nucleus, or not completed (nuclei on the borders) were hand-selected and excluded from the analysis. The Object Predictions and Object Identities were exported along with the CSV file, which was updated with the correct labels in the “User Labels” column. The “Predicted Class” column can also be used if ilastik’s neural network function is used to classify certain nuclear types.

After nuclei segmentation, sub-epidermal nuclei were filtered out using the “Variance of intensity” quantity. This calculates the inhomogeneity of the fluorescence signal across each nucleus. Mathematically, the variance of intensity *σ*_*i*_*^2^* for each nucleus *i* is calculated as:

(Equation 1) $$\sigma_{i}^{{}^{2}}=\frac{1}{n_{i}}\sum_{j=1}^{n_{i}}{\left(S_{j}-\mu_{i}\right)}^{2}\text{,}$$

where *n*_*i*_ is the number of voxels that correspond to the segmented nuclear volume of the nucleus *i*, *S*_*j*_ is the intensity of the fluorescent marker at voxel *j* for nucleus *i*, and *μ*_*i*_ is the mean intensity of the fluorescent marker of nucleus *i*. Plotting the variance of intensity in each nucleus for all nuclei gave two large peaks (Figure S2J). A threshold of 2^8.3^, which lies right in the trough of the two peaks, was used (Figure S2J, dashed white line). Sub-epidermal nuclei were defined to have a variance of intensity lower than this threshold. From our observations, these nuclei are sub-epidermal (Figure S2K). For the flow cytometry data, nuclei were filtered out that had a total *pML::H2B-mTFP* intensity lower than 2^9^ because these nuclei are most likely stomatal guard cell nuclei (Figure S2G).

#### Nuclear segmentation and data processing for *Drosophila melanogaster*

Following image acquisition, images were imported into Fiji/ImageJ and converted to a sum projection. This was then exported as a TIF file. The “2D Pixel Classification” workflow was used to create a pixel probability map for each image. After exporting the probability map, the “Object Classification [Inputs: Raw Data, Pixel Prediction Map]” workflow was used to conduct hysteresis thresholding for the nuclear segmentation using the smoothing parameters (1.0, 1.0), a core threshold of 0.6–0.8, and a final threshold of 0.5–0.7, depending on the image. All available nuclear features were calculated except “Skewness of intensity”, “Skewness of Intensity in neighborhood”, “Kurtosis of Intensity”, and “Kurtosis of Intensity in neighborhood”. For the uninjured and mild injury pylori, pyloric nuclei were manually selected that were either incomplete (e.g., partially out of frame) or belonged to a neighboring tissue and were removed from quantification. For the severely injured pylori and spermatids, all nuclei/spermatids that were complete and belonged to the tissue were selected by hand. Hand-selections were conducted by a single researcher and validated by another researcher. The Object Predictions and Object Identities were exported along with the CSV file, which was updated with the correct labels in the “User Labels” column.

The total Hoechst intensity (column name “Total Intensity” or “Total Intensity_0”, depending on the version of ilastik) was normalized by the spermatid intensities as in previous work.^26^ For each experiment, spermatids were imaged on the same slide as the fly tissue. Only those experiments in which we obtained at least 10 spermatids were used, where the median spermatid intensity was 2,000–6,000, and 90% of the spermatid nuclear intensity, when divided by the median spermatid intensity, was 0.5–1.5 (see Figure S4B). For experiments that met these conditions, the intensity of each pyloric nucleus was divided by the median spermatid intensity to create a “Normalized Hoechst Intensity” quantity, whereby a value of 2^0^ = 1 corresponded to 1C, 2^1^ corresponded to 2C, 2^2^ to 4C, and so on. Any experiments that did not satisfy the spermatid criteria were not considered.

#### Nuclear segmentation and data processing for human cardiomyocytes

Microscopy images were exported into an Imaris File format (.ims), and the nuclei were visualized in three dimensions using the ImarisCell module, where cardiomyocyte nuclei that were complete and not partially severed were identified (Imaris Version 8.2). Cardiomyocyte nuclei were selected by two researchers using both phalloidin and WGA labeling. The .ims files were imported into Fiji and were exported as TIF files for ilastik analysis. Not all organ donor samples were analyzed. The “2D Pixel Classification” workflow was used to create a pixel probability map for each image. Then, the “Object classification [Inputs: Raw Data, Pixel Prediction Map]” workflow was used to conduct hysteresis thresholding for the nuclear segmentation with the smoothing parameters (1.0, 1.0), a core threshold of 0.8, and a final threshold of 0.5. All available nuclear features were calculated. When using the Object Classification step, cardiomyocytes with complete nuclei, verified by the 3D rendering in ImarisCell, were labeled as “Cardiomyocyte nucleus” and were used for further analysis. The Object Predictions and Object Identities were exported, along with the CSV file, which was updated with the correct labels in the “User Labels” column.

### Quantification and statistical analysis

#### Gaussian mixture models

All ploidy predictions were performed using unsupervised Gaussian mixture models in the Scikit-learn Python package,^34^ which implements the expectation-maximization algorithm.^71^ In this work, either one- or two-dimensional Gaussians (1D or 2D) were used, depending on whether one (e.g., total nuclear signal intensity, also referred to as total signal intensity) or two nuclear features (e.g., total signal intensity and nuclear volume) were considered. Before performing the Gaussian mixture model, the data were logarithmically scaled by 2, which implied that each Gaussian would have a probability density function similar to a log-normal distribution with base 2. Although this is an unsupervised learning algorithm, the optimal fit was searched for by performing Gaussian mixture models while varying the number of components (i.e., the number of Gaussians added together to make the full Gaussian mixture) and the types of covariance matrices that were available in the Scikit-learn package: spherical (each component has a single variance), diagonal (each component has its own diagonal covariance matrix), full (each component has its own general covariance matrix), and tied (all components share the same general covariance matrix).

For the 1D Gaussian mixtures, a tolerance of 10^−5^, a maximum number of iterations of 10,000, 20 initializations, and the k-means++ initialization method were used. For the 2D Gaussian mixtures, a tolerance of 10^−7^, a maximum number of iterations of 5,000, 500 initializations, and the k-means++ initialization method were used. For the 1D Gaussian mixture for the PI staining and *pML::H2B-TFP* intensities, the spherical covariance matrix with 5 components with a tolerance of 10^−7^, maximum iterations of 5,000, 500 initializations, and k-means++ initialization method was used (Figure S2H). Such parameter values were set to avoid underfitting the data.

After identifying the optimal fit for each combination of components and covariance matrices, both the Akaike and Bayesian Information Criteria (AIC and BIC, respectively) were used to find the best fit given a certain covariance matrix.^34^^,^^35^^,^^72^ We use the definition used in the Scikit-learn package, i.e.,

(Equation 2) $$AIC=-2\;\log\left(\hat{L}\right)+2d\text{,}$$

(Equation 3) $$BIC=-2\;\log\left(\hat{L}\right)+\log\left(n\right)d\text{,}$$

where $\hat{L}$ is the maximum likelihood of the model, *d* is the number of parameters, and *n* is the number of samples (in this case, nuclei).^34^ This model was subsequently used after obtaining the best fit from the information criteria. The predict function was then used, which assigns which component each nucleus is likely to belong to by using the loglikelihood probabilities.

#### Error bars for ploidy histograms

To create the uncertainty bars for all ploidy histograms (e.g., Figure 2D), the following procedure was utilized, where *n* is the number of samples and *K* is the number of components computed from the Gaussian mixture model.

1. The log likelihood probabilities $\left\{p_{m}^{1},...,p_{m}^{K}\right\}$ for each nucleus *m* = {1, …,*n*} to belong to each component *k* = {1, …,*K*} were computed using the predict_proba function. Note that $\sum_{k=1}^{K}p_{m}^{k}=1$ for all *m*.
2. The maximum log likelihood probability for a nucleus *m* was defined as $p_{m}^{k_{\max}}$. This implies that the nucleus *m* was classified as part of the component *k*_*max*_. The proportions of nuclei that were classified into component *k*, i.e., all *m* such that *k*_*max*_ = *k*, were defined as the set {*V*^1^, …,*V*^*K*^}.
3. If $p_{m}^{k_{\max}}>0.8$, the classification was confident enough that *m* was classified into the component *k*_*max*_.
4. The number of nuclei classified into each component where $p_{m}^{k_{\max}}<0.8$ was calculated and defined $\left\{C_{out}^{1},...,C_{out}^{K}\right\}$ as the proportion of nuclei that could be classified to another component.
5. Lastly, all nuclei in which the second-largest log likelihood probability was greater than 0.2 were found. The number of nuclei that satisfied this condition for each component was calculated and $\left\{C_{in}^{1},...,C_{in}^{K}\right\}$ was defined as the proportion of nuclei that could be classified into one of these components.
6. Therefore, with the above considerations, the uncertainty bars were defined as those which spanned the interval $\left({V^{k}-C_{out}^{k},V}^{k}+C_{in}^{k}\right)$ for each component *k*.

## Supplemental information

Document S1. Figures S1–S6

Table S1. The exact values for all histograms plotted throughout the manuscript, related to Figures 2–4, S3, S5, and S6

Document S2. Article plus supplemental information
