# Supplementary material for: Spatial ploidy inference using quantitative imaging
Source: Cell Rep Methods. 2025 Dec 4;5(12):101249. doi: 10.1016/j.crmeth.2025.101249 (PMC12859483; doi:10.1016/j.crmeth.2025.101249)
Supplement: Document S1. Figures S1–S6 [file mmc1.pdf]

Cell Reports Methods, Volume *5*

Supplemental information

Spatial ploidy inference

using quantitative imaging

Nicholas J. Russell, Paulo B. Belato, Lilijana Sarabia Oliver, Archan Chakraborty, Adrienne H.K. Roeder, Donald T. Fox, and Pau Formosa-Jordan

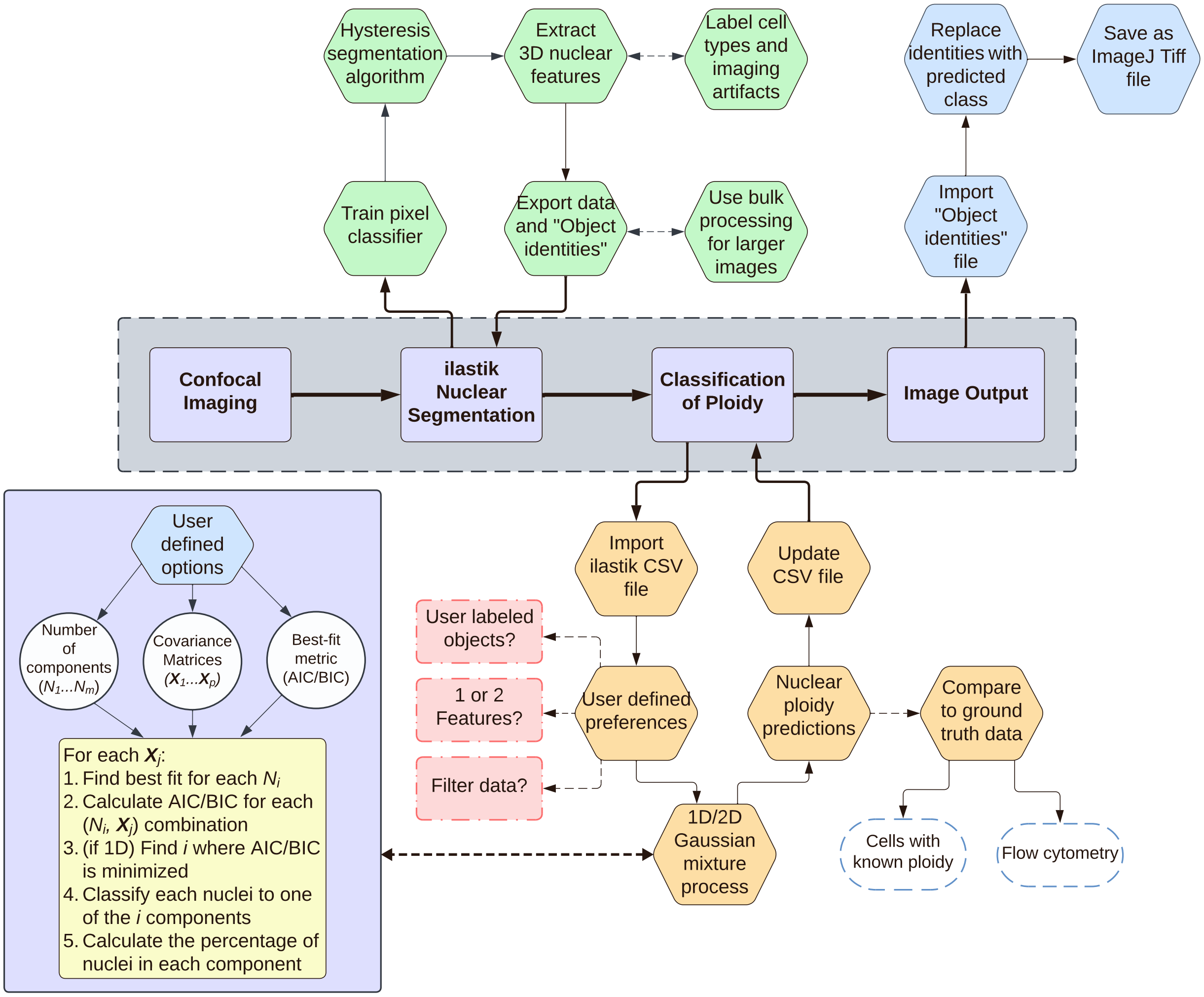

Figure S1: The image based spatial ploidy pipeline with Gaussian mixture model information. Related to Figure 1. The full iSPy methodology, which includes the Gaussian mixture implementation. See Results and STAR Methods for further details.

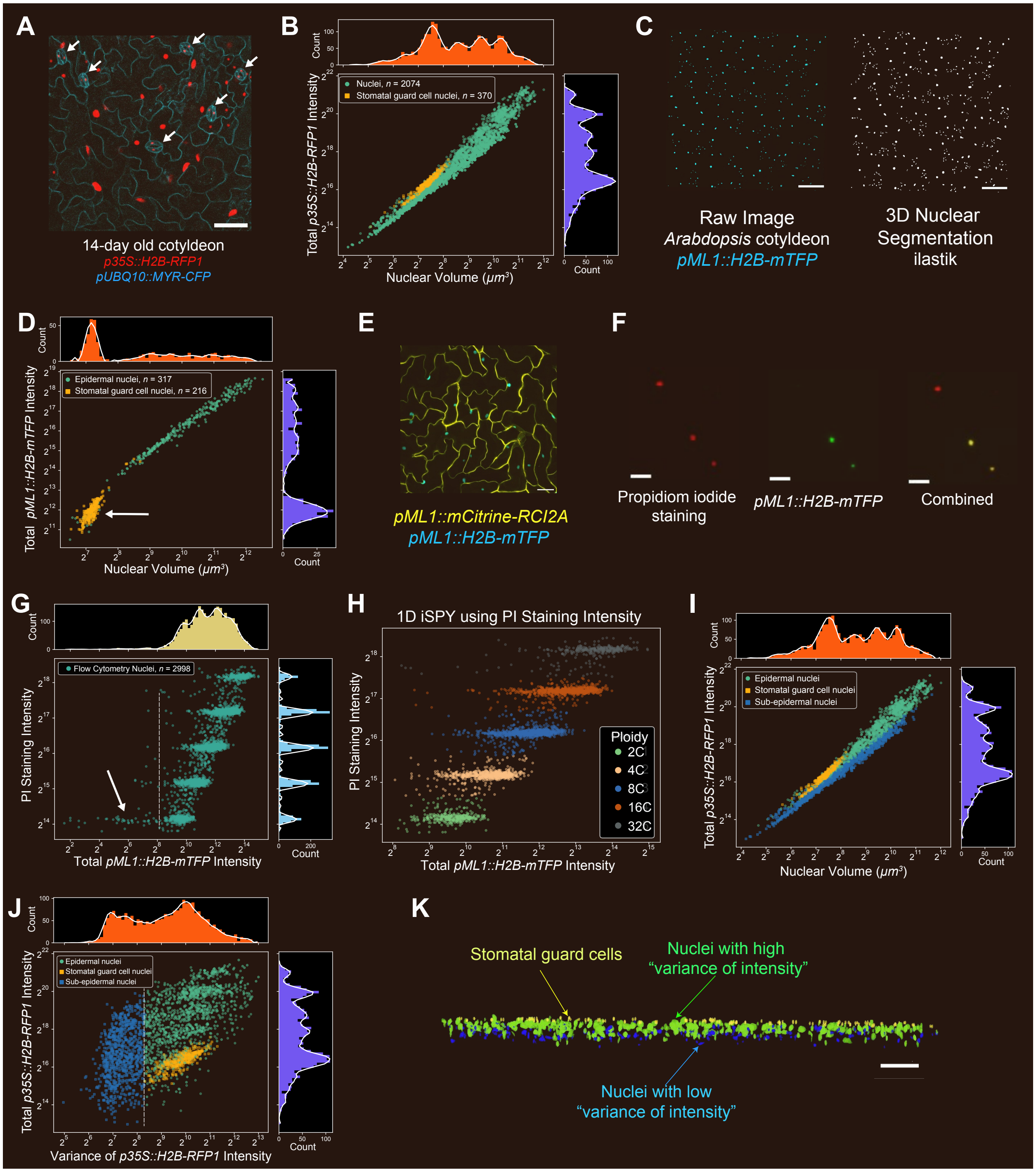

<u>Figure S2: *pML1::H2B-mTFP* can identify epidermal cells and stomatal guard cells, and subepidermal cells have low variance of intensity.</u> Related to Figure 2 and STAR Methods sections “Flow cytometry for *Arabidopsis thaliana*” and “Nuclear segmentation and data processing for *Arabidopsis thaliana*”. (**A**) Representative confocal image of 14-day-old *Arabidopsis* cotyledon tissue expressing the nuclear marker *p35S::H2B-RFP1* and the membrane marker *pUBQ10::MYR-CFP*. Arrows denote the stomatal complexes, which are readily identifiable due to their unique cellular morphology. The membrane marker was used for the classification of stomatal guard cell nuclei. Scale bar = 25 μm. **(B)** Scatterplot of the nuclear volume and total *p35S::H2B-RFP1* intensity of the ilastik-segmented nuclei (green) and stomatal guard cell nuclei (yellow), including corresponding histograms of the total

p35S::H2B-RFP1 intensity (purple) and nuclear volume (orange) with a smoothed Savitzky–Golay filter (white line, only for illustrative purposes). **(C)** Left: representative confocal image of *pML1::H2B-mTFP* in a 14-day-old cotyledon. Right: nuclear segmentation of the left image performed in ilastik. Note that segmentation was performed in three dimensions. Scale bars = 100 µm. **(D)** Scatterplot of the nuclear volume and total *p35S::H2B-RFP1* intensity of the segmented nuclei (epidermal nuclei, green circles; stomatal guard cells, yellow squares), including representative histograms of the total *p35S::H2B-RFP1* intensity (purple) and nuclear volume (orange) with a smoothed Savitzky–Golay filter (white line, only for illustrative purposes). Note the cluster of stomatal guard cells (arrow) and the lack of clusters in the epidermal cells, contrary to what was observed with *p35S::H2B-RFP1* (see Figure 2B, STAR Methods). **(E–H)** Data related to the flow cytometry analysis (see STAR Methods for experimental information). **(E)** Representative image of a 14-day cotyledon used for flow cytometry tagged with epidermal-specific cell membrane fluorescence marker *pML1::mCirtrine-RCI2A* (yellow) and the epidermal-specific nuclear marker *pML1::H2B-mTFP* (blue). Scale bar = 25 µm. **(F)** A confocal image of nuclei from a *pML1::mCirtrine-RCI2A* × *pML1::H2B-mTFP* cotyledon stained with propidium iodide (PI) (left). Note that not all cells express *pML1::H2B-mTFP* (middle, right), which allows for the identification of epidermal cells. Scale bars = 20 µm. **(G)** Scatterplot of the total *pML1::H2B-mTFP* intensity and total PI staining intensity, including representative histograms of the total *pML1::H2B-mTFP* intensity (yellow) and total PI staining intensity (blue) with a smoothed Savitzky–Golay filter (white line, only for illustrative purposes). Only cells that express *pML1::H2B-mTFP* are shown (i.e., epidermal cells). Note the cluster of stomatal guard cells (arrow) similar to the confocal imaging data in (C). We use a threshold of 2^9 to remove all stomatal guard cells from the calculations (white dashed line). **(H)** Classification of ploidy from the flow cytometry data using a 1D Gaussian mixture with five components and full covariance matrices on the total intensity of the PI staining. Percentages of nuclei in each ploidy class can be found in Figure 2D (green bar) and Table S1. **(I)** Scatterplot of the nuclear volume and variance of the *p35S::H2B-RFP1* intensity of the segmented nuclei, including representative histograms of the total *p35S::H2B-RFP1* intensity (purple) and variance of the *p35S::H2B-RFP1* intensity (orange) with a smoothed Savitzky–Golay filter (white line, only for illustrative purposes). Note the bi-modal distribution in the histogram of the variance of the *p35S::H2B-RFP1* intensity. A threshold of $2^{8.3}$ (white dashed line) was used, and any cells beneath this threshold were classified as sub-epidermal cells (blue squares). **(J)** Scatterplot of the nuclear volume and total *p35S::H2B-RFP1* intensity of the segmented nuclei (epidermal nuclei, green circles; stomatal guard cells, yellow squares; sub-epidermal nuclei, blue squares), including representative histograms of the total *p35S::H2B-RFP1* intensity (purple) and nuclear volume (orange) with a smoothed Savitzky–Golay filter (white line, only for illustrative purposes). **(K)** A representative snapshot of segmented nuclei, with the low-variance of intensity nuclei shaded in blue, and in green and yellow for high-variance of intensity nuclei and stomatal guard cells, respectively. Note that nuclei with a higher variance of intensity are above those with a lower value in the scatterplot. Scale bar = 100 µm.

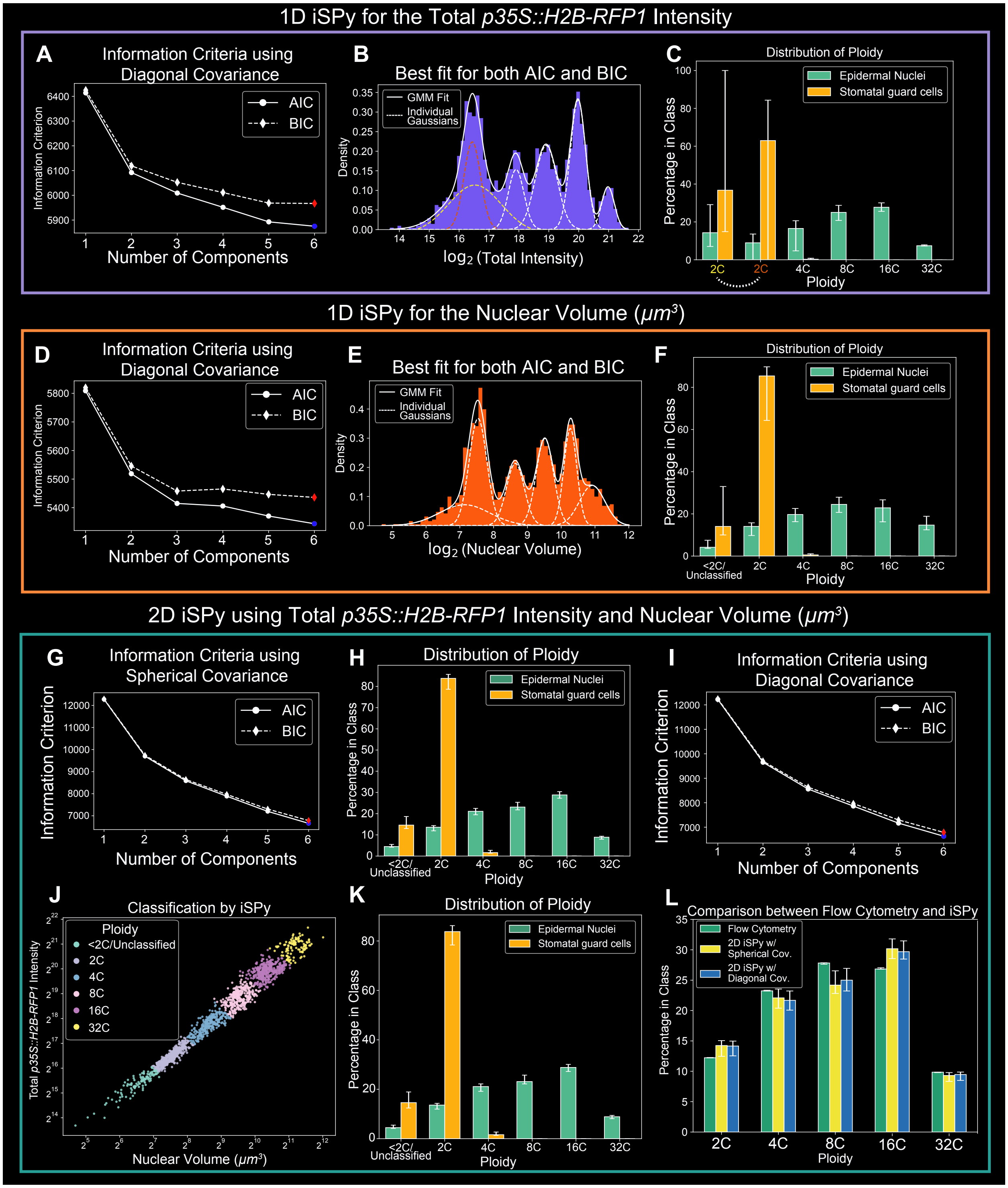

Figure S3: 1D and 2D Gaussian mixtures for *Arabidopsis* cotyledons predict six components. Related to Figure 2 and STAR Methods section "Gaussian mixture models". **(A–C)** A 1D Gaussian mixture for the total *p35S::H2B-RFP1* intensity (see STAR Methods for details). **(A)** Values of AIC (solid line) and BIC (dashed line) as the number of components is varied while using the diagonal covariance matrices. The blue and red colored points at six components represent the lowest AIC and BIC, respectively, and therefore the best fit. **(B)** The best fit using the 1D Gaussian mixture displaying the histogram (purple), the full Gaussian mixture fit (solid white line), and the six components (dashed lines) that sum up to equal the best fit. The two colored Gaussians (yellow and orange)

represent 2C cells, and the subsequent Gaussians represent 4C, 8C, 16C, and 32C cells, respectively. **(C)** The proportion of stomatal guard cells (yellow) and epidermal cells (green) predicted to be in each ploidy class. Note the two 2C classes, which correspond to the colored Gaussians in (B). See Table S1 for exact values. **(D–F)** A 1D Gaussian mixture for the nuclear volume. **(D)** Values of AIC (solid line) and BIC (dashed line) as the number of components is varied while using the diagonal covariance matrices. The blue and red colored points at 6 components represent the lowest AIC and BIC, respectively, and, therefore, the best fit. **(E)** The best fit using the 1D Gaussian mixture displaying the histogram (orange), the full Gaussian mixture fit (solid white line), and the six components (dashed lines) that sum up to equal the best fit. The Gaussians represent the <2C/Unclassified, 2C, 4C, 8C, 16C, and 32C cells, respectively. **(F)** The proportion of stomatal guard cells and epidermal cells predicted to be in each ploidy class. Note the high number of stomatal guard cells in the 2C group compared to the unclassified and 4C groups. See Table S1 for exact values. Uncertainty bars represent nuclei that may be classified incorrectly (log likelihood probability less than 0.8) and nuclei that could be classified in another component (log likelihood probability greater than 0.2) (see STAR Methods). **(G, H)** A 2D Gaussian mixture using both total *p35S::H2B-RFP1* intensity and spherical covariance found in Figures 2C–2F. **(G)** Values of AIC (solid line) and BIC (dashed line) as the number of components is varied while using the spherical covariance matrices. The blue and red colored points at six components represent the lowest AIC and BIC, respectively and, therefore, the best fit. **(H)** The proportion of stomatal guard cells (yellow) and epidermal cells (green) predicted to be in each ploidy class. **(I–K)** A 2D Gaussian mixture using both total *p35S::H2B-RFP1* intensity and diagonal covariance. See Table S1 for exact values. **(I)** Values of AIC (solid line) and BIC (dashed line) as the number of components is varied while using the diagonal covariance matrices. The blue and red colored points at 6 components represent the lowest AIC and BIC, respectively, and therefore the best fit. **(J)** iSPy prediction for each epidermal nucleus using the 2D Gaussian mixture with six components and spherical covariance matrix. **(K)** The proportion of stomatal guard cells (yellow) and epidermal cells (green) predicted to be in each ploidy class. **(L)** The comparison of the percentage of epidermal nuclei predicted in each ploidy class between flow cytometry (green, Figures S2G–S2J), 2D iSPy Gaussian mixture with spherical covariance (yellow, (G, H) and Figure 2C), and the 2D iSPy Gaussian mixture with diagonal covariance (blue, (I–K)). Uncertainty bars in all plots represent nuclei that may be classified incorrectly (log likelihood probability less than 0.8) and nuclei that could be classified in another component (log likelihood probability greater than 0.2) (see STAR Methods).

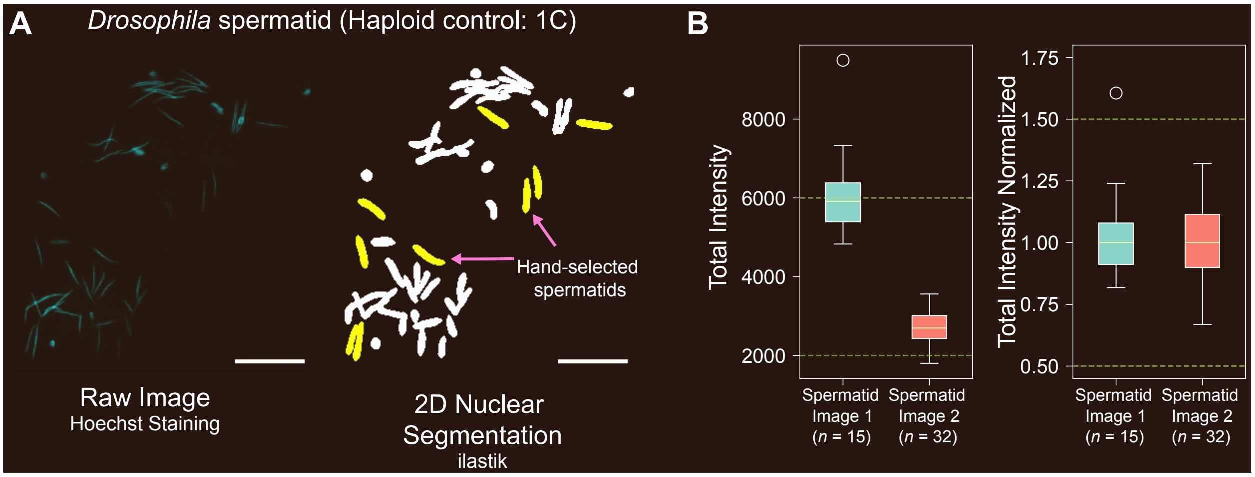

Figure S4: Quantification of haploid sperm nuclei from *Drosophila* provides a control. Related to STAR Methods section "Nuclear segmentation and data processing for *Drosophila melanogaster*." **(A)** Left: representative sum-projection of a confocal image of *Drosophila* spermatids stained with Hoechst. Right: segmented sperm nuclei using ilastik. Yellow nuclei signify hand-selected sperm that were complete and unobstructed (see STAR Methods). Scale bars = 20 µm. **(B)** Example quantification of two spermatid images from the same experiment and slide. Left: total intensity of hand-selected spermatids from two different images. Both have sample sizes larger than $n = 10$ and medians between 2,000 and 6,000 (dashed green lines). Right: normalized total intensity of both images. All data points were divided by the median total intensity from each image. We mandate that at least 90% of normalized total intensity values are between 0.5 and 1.5 (dashed green lines). We found that 14/15 (93%) of the spermatids from the first image fit this criterion, and 32/32 (100%) of spermatids from the second image fit this criterion. See STAR Methods for a more detailed explanation.

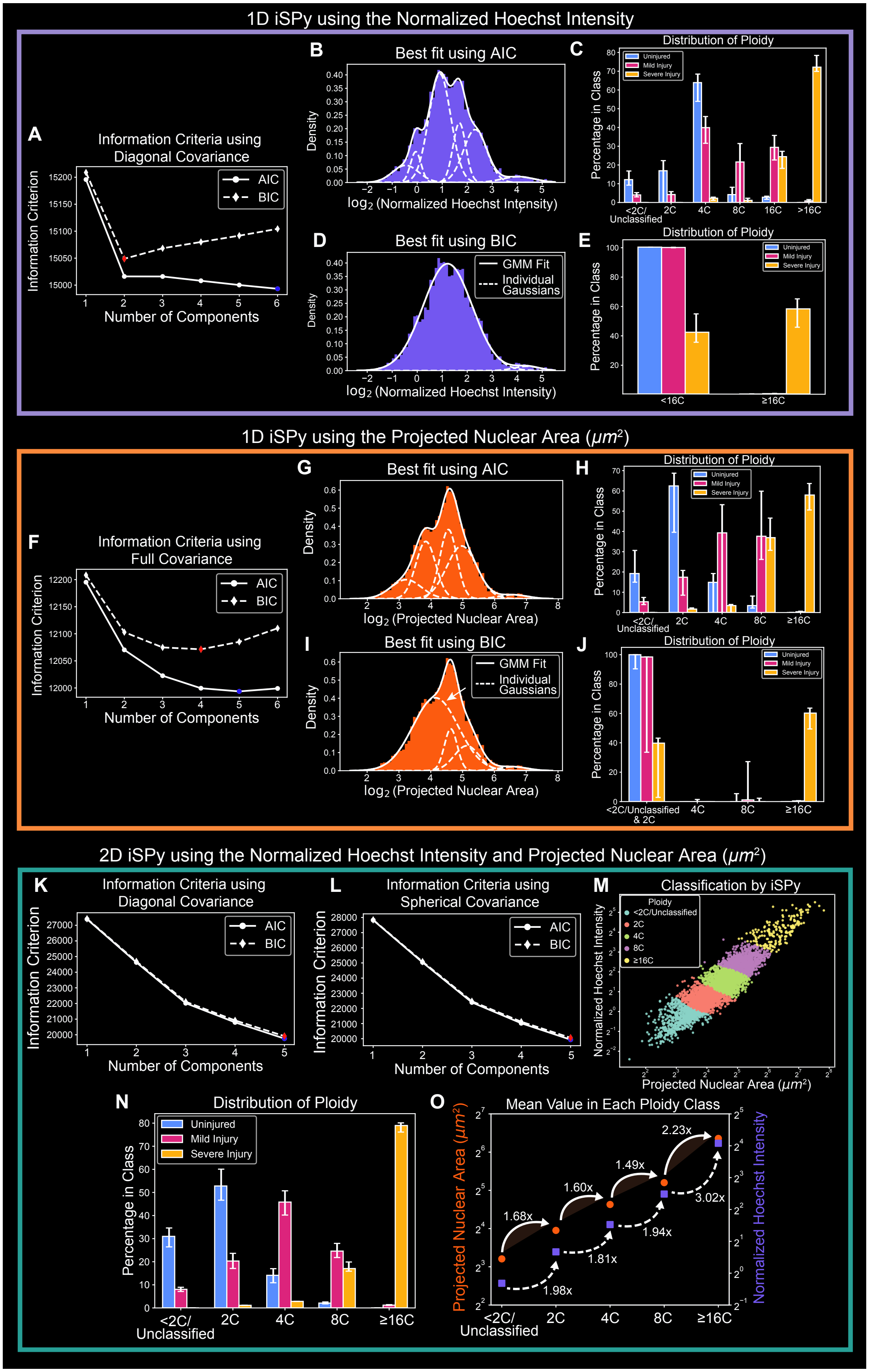
1D iSPy using the Normalized Hoechst Intensity
A
Information Criteria using Diagonal Covariance
Information Criterion
Number of Components
AIC
BIC
B
Best fit using AIC
Density
log2 (Normalized Hoechst Intensity)
C
Distribution of Ploidy
Percentage in Class
Uninjured
Mild Injury
Severe Injury
<2C/ Unclassified
2C
4C
8C
16C
>16C
D
Best fit using BIC
GMM Fit
Individual Gaussians
E
<16C
≥16C
1D iSPy using the Projected Nuclear Area (μm²)
F
Information Criteria using Full Covariance
G
Best fit using AIC
log2 (Projected Nuclear Area)
H
I
Best fit using BIC
J
<2C/Unclassified & 2C
2D iSPy using the Normalized Hoechst Intensity and Projected Nuclear Area (μm²)
K
Information Criteria using Diagonal Covariance
L
Information Criteria using Spherical Covariance
M
Classification by iSPy
Ploidy
Normalized Hoechst Intensity
Projected Nuclear Area (μm²)
N
Distribution of Ploidy
O
Mean Value in Each Ploidy Class
Projected Nuclear Area (μm²)
1.68x
1.60x
1.49x
2.23x
1.98x
1.81x
1.94x
3.02x

<u>Figure S5: AIC and BIC produce different best fits for *Drosophila* pyloric cells.</u> Related to Figure 3 and STAR Methods sections "Gaussian mixture models" and "Nuclear segmentation and data processing for *Drosophila melanogaster*." **(A–E)** A 1D Gaussian mixture for the normalized Hoechst intensity (see STAR Methods). **(A)** Values of AIC (solid line) and BIC (dashed line) as the number of components is varied while using the full covariance matrices. The blue and red colored points represent the lowest AIC (five components) and BIC (four components), respectively. **(B)** The best fit of the 1D Gaussian mixture using AIC displaying the histogram (purple), the full Gaussian mixture fit (solid white line), and the six components (dashed lines). **(C)** The proportion of uninjured pyloric cells (blue), mildly injured pyloric cells (red), and severely injured pyloric cells (yellow) in each predicted ploidy class: <2C/Unclassified, 2C, 4C, 8C, 16C, and >16C. **(D)** The best fit of the 1D Gaussian mixture using BIC displaying the histogram (purple), the full Gaussian mixture fit (solid white line), and the two components (dashed lines). **(E)** The proportion of uninjured pyloric cells (blue), mildly injured pyloric cells (red), and severely injured pyloric cells (yellow) in each predicted ploidy class: <16C and ≥16C. **(F–J)** A 1D Gaussian mixture for the projected nuclear area (see STAR Methods). **(F)** Values of AIC (solid line) and BIC (dashed line) as the number of components is varied while using the spherical covariance matrices. The blue and red colored points represent the lowest AIC (five components) and BIC (four components), respectively. **(G)** The best fit of the 1D Gaussian mixture using AIC displaying the histogram (orange), the full Gaussian mixture fit (solid white line), and the five components (dashed lines). **(H)** The proportion of uninjured pyloric cells (blue), mildly injured pyloric cells (red), and severely injured pyloric cells (yellow) in each predicted ploidy class: <2C/Unclassified, 2C, 4C, 8C, and ≥16C. **(I)** The best fit of the 1D Gaussian mixture using BIC displaying the histogram (orange), the full Gaussian mixture fit (solid white line), and the four components (dashed lines). Note the large component (arrow) marks cells between <2C/Unclassified and 8C. **(J)** The proportion of uninjured pyloric cells (blue), mildly injured pyloric cells (red), and severely injured pyloric cells (yellow) in each predicted ploidy class: <2C/Unclassified & 2C, 4C, 8C, and ≥16C. See Table S1 for exact values for (C), (E), (H), and (J). All uncertainty bars represent nuclei that may be classified incorrectly (log likelihood probability less than 0.8) and nuclei that could be classified in another component (log likelihood probability greater than 0.2) (see STAR Methods). **(K–L)** Values of AIC (solid line) and BIC (dashed line) as the number of components is varied while using the **(K)** diagonal or **(L)** spherical covariance matrices. The blue and red colored points at five components represent the lowest AIC and BIC, respectively, and therefore the best fit. The best fit for (K) is shown in Figure 3C. **(M)** iSPy prediction for each pyloric nucleus using the 2D Gaussian mixture with five components and spherical covariance matrix corresponding to ploidies of <2C/Unclassified, 2C, 4C, 8C, and ≥16C. **(N)** The proportion of pyloric cells in each ploidy class predicted from the 2D Gaussian mixture in (M) by severity of injury (uninjured, blue; mild injury, red; severe injury, yellow). Uncertainty bars represent nuclei that may be classified incorrectly (log likelihood probability less than 0.8) and nuclei that could be classified in another component (log likelihood probability greater than 0.2) (see STAR Methods). See Table S1 for exact values. **(O)** The mean of the normalized Hoechst intensity (purple squares, right axis) and projected nuclear area (orange circles, left axis) with the fold increase to the next ploidy class using the 2D Gaussian mixture in (M).

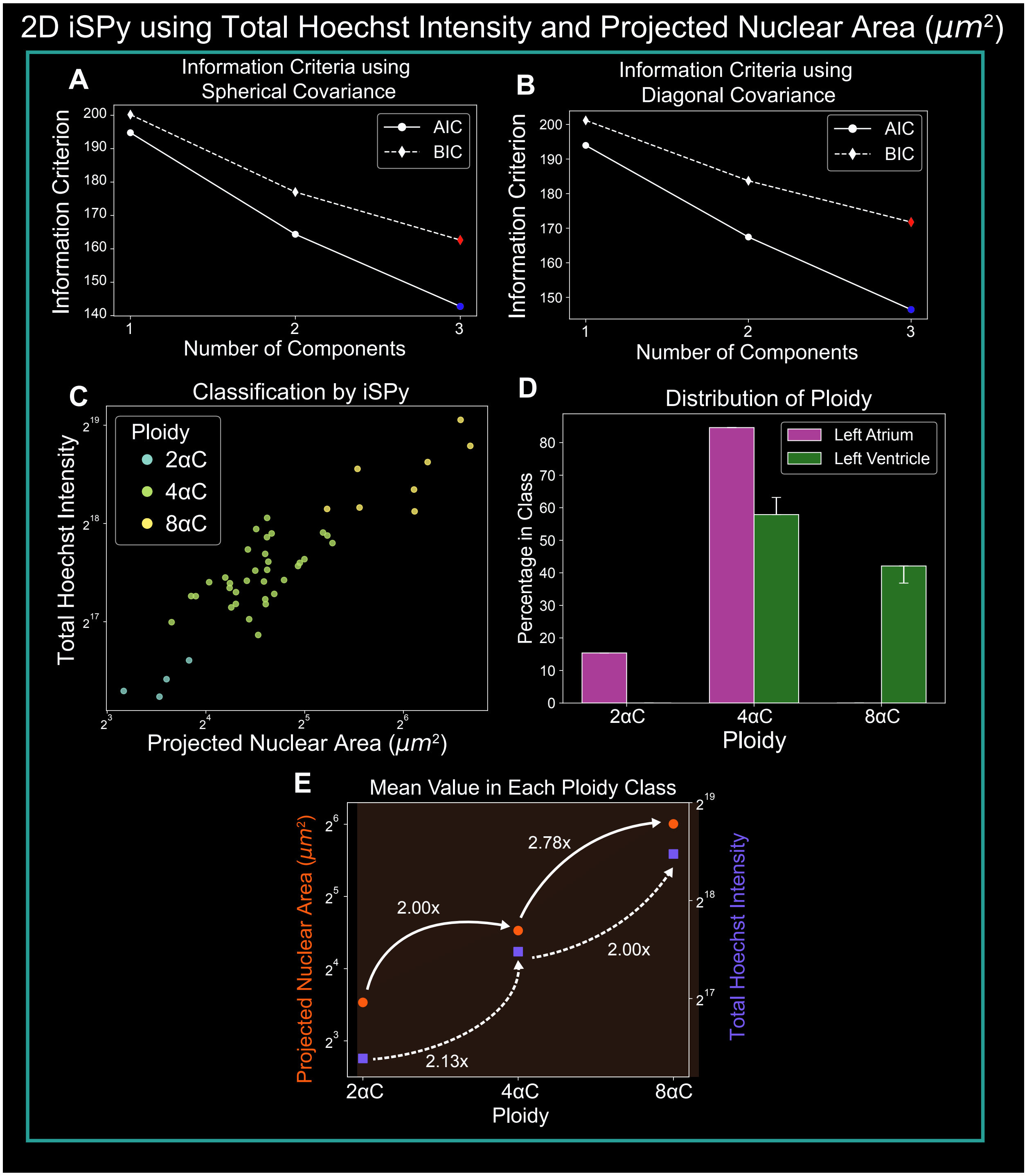

<u>Figure S6: Using either spherical or diagonal covariance matrices predicts three ploidy classes for human cardiomyocytes.</u> Related to Figure 4. **(A, B)** Values of both information criteria AIC (solid line) and BIC (dashed line) as the number of components is varied while using the **(A)** spherical or **(B)** diagonal covariance matrices. The blue and red colored points at three components represent the lowest AIC and BIC, respectively, and therefore the best fit. The best fit for (A) is shown in Figure 4C. **(C)** iSPy prediction for each pyloric nucleus using the 2D Gaussian mixture with three components and diagonal covariance matrix corresponding to ploidies of 2αC, 4αC, and 8αC. **(D)** The proportion of cardiomyocytes in each ploidy class predicted from the 2D Gaussian mixture in (C) by heart chamber (left atrium, purple; left ventricle, green). See Table S1 for exact values. Uncertainty bars

represent nuclei that may be classified incorrectly (log likelihood probability less than 0.8) and nuclei that could be classified in another component (log likelihood probability greater than 0.2) (see STAR Methods). **(E)** The mean of the Hoechst intensity (purple squares, right axis) and projected nuclear area (orange circles, left axis) with fold-increase to the next ploidy class using three components and diagonal covariance.
